# Can Bacteriophages Be Effectively Utilized for Disinfection in Animal-Derived Food Products? A Systematic Review

**DOI:** 10.3390/pathogens14030291

**Published:** 2025-03-16

**Authors:** Rafail Fokas, Zoi Kotsiri, Apostolos Vantarakis

**Affiliations:** Department of Public Health, Medical School, University of Patras, 26504 Patras, Greece; zoikotsiri@upatras.gr

**Keywords:** bacteriophages, food pathogens, food safety, disinfection, food quality, food hygiene

## Abstract

Food safety is a paramount public health concern, particularly with the rise of antimicrobial-resistant bacteria. This systematic review explores the efficacy of bacteriophages as a novel and environmentally sustainable approach to controlling multi-resistant and non-resistant bacterial pathogens in animal-derived food products. Following PRISMA guidelines, data from multiple studies were synthesized to evaluate bacteriophage applications across diverse food matrices, including beef, poultry, seafood, and dairy. The findings highlight significant variability in bacteriophage efficacy, influenced by factors such as food matrix properties, bacterial strains, and application methods. Phage cocktails and their combination with thermal treatments consistently demonstrated superior bacterial reduction compared to single-phage applications, which yielded variable results. Interestingly, the absence of a clear dose-response relationship underscores the need for a more detailed understanding of phage-host interactions and environmental influences. This review addresses a critical gap in the literature by advocating for matrix-specific, targeted phage applications over generalized approaches. Additionally, it underscores the transformative potential of bacteriophages as sustainable alternatives to chemical disinfectants in modern food safety practices. These insights provide a framework for future research aimed at optimizing bacteriophage efficacy and scaling their application in real-world food production systems.

## 1. Introduction

The correlation between food consumption and human ailments was acknowledged in antiquity, with Hippocrates (460 B.C.) being among the first to document the profound link between dietary intake and human health [1]. The term “food safety” refers to the scientific and hygienic practices applied during food preparation, handling, and storage, aiming to prevent foodborne illnesses and maintain the nutritional integrity of the food [2]. Food hygiene and safety practices are subject to substantial control over the origin of the food, the food label, the additives, the residues of phytopharmaceuticals as well as the microbial load they carry [3]. Ensuring adequate access to safe and nutritious food is fundamental for sustaining human life and fostering optimal health. Consumption of unsafe food, contaminated with pathogenic microorganisms, viruses, parasites, or toxic chemicals, contributes to the incidence of over 200 diseases [4], spanning from gastrointestinal illnesses to various forms of cancer. This creates a harmful cycle of illness and malnutrition, with a disproportionate impact on vulnerable groups like infants, young children, the elderly, and those with weakened immune systems. The World Health Organization estimates that contaminated food leads to 600 million cases of illness and 420,000 deaths each year. Alarmingly, 30% of these fatalities are children under five, and low- and middle-income countries suffer a $110 billion loss annually in productivity and healthcare costs [5]. In 2022, according to European Center for Disease Prevention and Control (ECDC) reports, the number of cases, hospitalizations, and deaths from foodborne causes in Europe was much higher compared to 2021 [6].

Growth in global meat protein consumption over the next decade is projected to increase by 14% by 2030 compared to the average of the 2018–2020 base period, driven by income and population growth [7]. According to the CDC [8], although meats are blamed for fewer illnesses than other food categories, they still account for 29% of foodborne illness deaths. Poultry accounted for most deaths (19%). Many of these were caused by Listeria and Salmonella infections. Food and Agriculture Organization FAO [9] points out that one-third of the global burden of foodborne diseases is related to the consumption of contaminated terrestrial food of animal origin (TASF). Despite the fact that the vegan diet is flourishing, the need for foods of animal origin remains, emphasizing the importance of methods of protection and hygiene in these foods. However, even if the consumption of animal-based foods is set aside in the long term and the consumption of meat substitutes increases, the need for safe food remains the same. Recent studies indicate that pathogens commonly found in animal-based foods are also capable of colonizing meat substitutes [10,11].

The foundation of food safety lies in the intricate interplay between the food industry and the consumer market, followed by the dynamic between market forces and consumer behaviors. Foodborne illnesses extend beyond industry practices, often stemming from mishandling by consumers themselves, including inadequate product storage or preparation. It is imperative that relevant health or governmental authorities, along with educational institutions, undertake the crucial task of consumer education. This aspect remains largely unregulated, contingent solely upon consumer choices. Within the food sector, strict adherence to hygiene standards is not only legally mandated but also constitutes a fundamental obligation for all entities involved in public health. Accordingly, robust quality assurance systems and certifications, such as ISO and HACCP, are established prerequisites. While these protocols partially address the realm of food safety, outbreaks of foodborne illnesses persist. Moreover, the emergence of heightened antibiotic resistance among certain bacteria, coupled with their resilience to conventional cleaning agents, underscores the urgent need for sustainable and environmentally friendly disinfection methodologies that benefit both food products and human health.

For numerous decades, bacteriophages have been extensively researched for their targeted antibacterial properties. Bacteriophages, also referred to as phages, are viral entities that infiltrate and replicate within bacterial cells [12]. They exhibit either a simple or complex structure and proliferate within host bacteria by injecting their genetic material into the cytoplasm. Notably, their impact on bacteria is highly selective, demonstrating precision in targeting specific bacterial strains. Widely distributed, it is estimated that approximately 10^31^ bacteriophages exist globally [13]. Detected with regularity in environmental samples, bacteriophages are also an integral component of the human body’s microbiome [14]. Their potential to combat antibiotic-resistant bacteria through phage therapy has attracted significant attention. Moreover, beyond medicinal applications, bacteriophages are under investigation for their prospective role in enhancing food safety measures, presenting an environmentally sustainable alternative to chemical disinfectants [15]. Since 2006, the FDA has approved several phage-based products for use in cheese and meat processing [16]. Their utility spans across various stages of food production, serving as both a preventive measure against contamination and a means of disinfection following contamination events. Upon introduction, bacteriophages infect and proliferate within target bacterial populations over extended durations. Consequently, they contribute to safeguarding food quality and safety throughout the production, processing, and preservation stages, including storage on supermarket shelves. Notably, in terms of preservation, bacteriophages offer a potential alternative to harmful food additives such as nitrites and nitrates commonly used in cured meats, which have been associated with a substantial proportion of colon cancer cases. However, caution must be exercised in the utilization of phages in fermented products involving lactic acid bacteria, as inadvertent disruption of the beneficial bacterial fermentation culture may occur. Nevertheless, with careful application, such adverse effects can be mitigated [17].

This systematic study investigates the promising potential of bacteriophages as a cutting-edge method for combating both multi-resistant and non-resistant bacteria in animal-derived foods. It investigates how naturally existing viral agents could transform food safety and quality, offering a new viewpoint on sustainable disinfection procedures. Addressing a fundamental gap in current scientific knowledge, this work aims to motivate a move away from traditional, frequently hazardous pharmaceutical therapies. By replacing them with eco-friendly bacteriophage applications, we pave the way for innovations that could not only preserve public health but also change the food industry’s attitude to hygiene and sustainability. The findings of this study could transform current approaches to guaranteeing safe food consumption, setting the groundwork for future developments that reconcile food safety with environmental consciousness.

## 2. Materials and Methods

**Figure 1:**
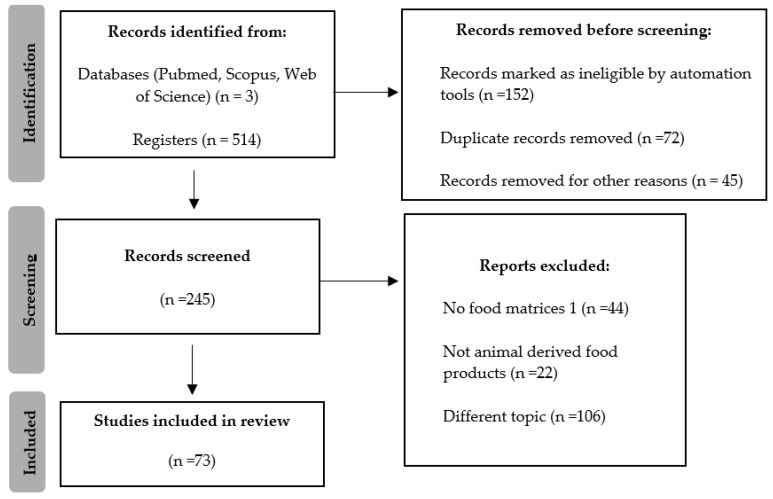
Prisma flow diagram.

In this investigation, adherence to the [18] PRISMA (Preferred Reporting Items for Systematic Reviews and Meta-Analyses) statement protocol was rigorously observed throughout all phases, encompassing literature retrieval, study curation, and analytical procedures (refer to Figure 1). This systematic review has been registered in the Open Science Framework (OSF) under the following link: https://osf.io/t36hd/ (accessed on 4 March 2025).

In the initial stage, all study designs were incorporated regardless of their publication date. The literature retrieval process was conducted without imposing language restrictions, provided that an English abstract containing pertinent information was available. The criteria for inclusion and exclusion were delineated as follows: The inclusion criteria for this review encompassed studies that employed lab-based techniques within food matrices, focusing specifically on animal-derived food products. Additionally, the review considered studies involving multi-resistant bacteria, antimicrobial resistance bacteria, or pathogenic bacteria, if they were published in English. Moreover, studies were required to demonstrate an impact on food safety. Conversely, studies were excluded if they involved non-lab-based techniques, or if their primary focus was on drugs or medicine rather than food safety. Research that did not pertain to animal-derived food products was also excluded, as were studies concerning viruses, fungi, or non-food pathogenic bacteria.

### 2.1. Information Sources and Literature Research

A comprehensive review of three significant online databases was used to undertake a complete assessment of empirical research published over the last five years, specifically from 2019 onwards: Scopus (Elsevier), Web of Science (Clarivate), and PubMed (NCBI). The literature search was conducted simultaneously on all three search sites in February 2024. We used the following search terms (adapted for each database):

((BACTERIOPHAGES OR PHAGE THERAPY OR PHAGE-BASED TREATMENT OR PHAGE APPLICATION OR PHAGE-TECHNOLOGY OR PHAGE CONTROL) AND (DISINFECTION OR DECONTAMINATION OR SANITATION OR ANTIMICROBIAL OR PATHOGEN CONTROL OR MICROBIAL REDUCTION OR FOOD SAFETY OR FOOD SANITATION) AND (FOODBORNE PATHOGENS OR FOOD CONTAMINATION OR FOOD ILLNESS OR FOOD SAFETY MEASURES) AND (MEAT PRODUCTS OR DAIRY PRODUCTS OR POULTRY PRODUCTS OR SEAFOOD PRODUCTS OR FOOD PROCESSING OR FOOD PRESERVATION OR FOOD INDUSTRY)).

### 2.2. Study Selection

The selection process for studies to be included in this systematic review was independently carried out by three reviewers (R.F., Z.K., and A.V.). Three independent reviewers screened all records and full-text reports for eligibility. The selection process was conducted through discussion among all reviewers to reach a consensus. Any disagreements were resolved collectively. Each reviewer worked separately to extract relevant information from the included studies. Any discrepancies in the extracted data were discussed and resolved through group consensus. Duplicate publications were identified and managed using Mendeley to ensure each article was considered only once. An initial screening of titles and abstracts was performed according to predefined inclusion and exclusion criteria, and potentially relevant articles underwent a more thorough full-text review. During this process, eligibility was independently assessed against set criteria to finalize the list of included studies. A total of 73 suitable articles [19,20,21,22,23,24,25,26,27,28,29,30,31,32,33,34,35,36,37,38,39,40,41,42,43,44,45,46,47,48,49,50,51,52,53,54,55,56,57,58,59,60,61,62,63,64,65,66,67,68,69,70,71,72,73,74,75,76,77,78,79,80,81,82,83,84,85,86,87,88,89,90,91] (Table S1) were identified and further categorized by host-bacteria, matrix, and method, resulting in 114 distinct outcomes due to some studies providing multiple data points. The effect measures used to assess bacteriophage efficacy included log reduction (log CFU/g or log CFU/mL), mean bacterial reduction, percentage reduction in bacterial counts, and comparative survival rates under different conditions. These measures were extracted as reported in the original studies and synthesized accordingly. A detailed presentation of the results is provided in the respective section. Bar charts were generated to represent country percentages, while stacked bar charts illustrated the distribution of methods across bacteria and matrices. Following further refinement, the dataset was narrowed to 89 rows, focusing on comparing log reductions across different categories, leading to the generation of box and scatter plots. Finally, the dataset was reduced to 45 rows, allowing for statistically meaningful dose comparisons of bacteria and phages, as well as log reduction.

### 2.3. Statistical Analysis

Multiple statistical methods were used to comprehensively examine the final dataset of 45 rows. First, normality tests, including the Kolmogorov-Smirnov and Shapiro-Wilk tests, were applied to evaluate the distribution characteristics of the variables: bacterial dose, phage dose, and log reduction. To further highlight the data distribution, QQ plots were created, which provided visual confirmation of the skewness and deviations, which were most visible in the distribution tails. Given the variables’ non-normal distribution, Spearman’s rank-order correlation was then used to analyze their associations. The purpose of this research was to assess whether or not there are statistically significant correlations between bacterial dosage, phage dose, and log decreases. All statistical analyses were performed using IBM SPSS Statistics 29.0 software (IBM Corp., Chicago, IL, USA).

## 3. Results

**Figure 2:**
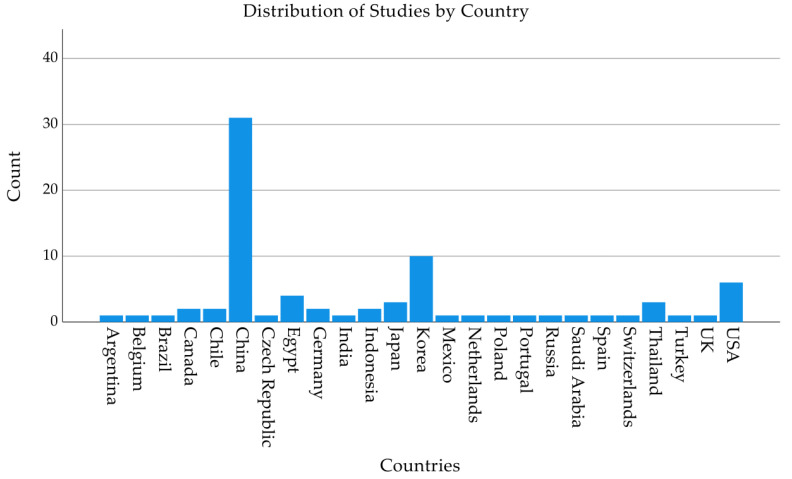
Bar Chart Showing the Geographic Distribution of Studies on Bacteriophage Use.

The geographical distribution of studies included in this systematic review reveals significant disparities. As shown in Figure 2, China leads with the highest number of published studies, exceeding 30, highlighting its substantial research activity in the field of bacteriophage application for the disinfection of animal-derived food products. Following China are the United States and Korea, with a moderate contribution from countries such as Canada and the United Kingdom. In contrast, several countries, including Brazil, Spain, and Saudi Arabia, contributed to less studies.

**Figure 3:**
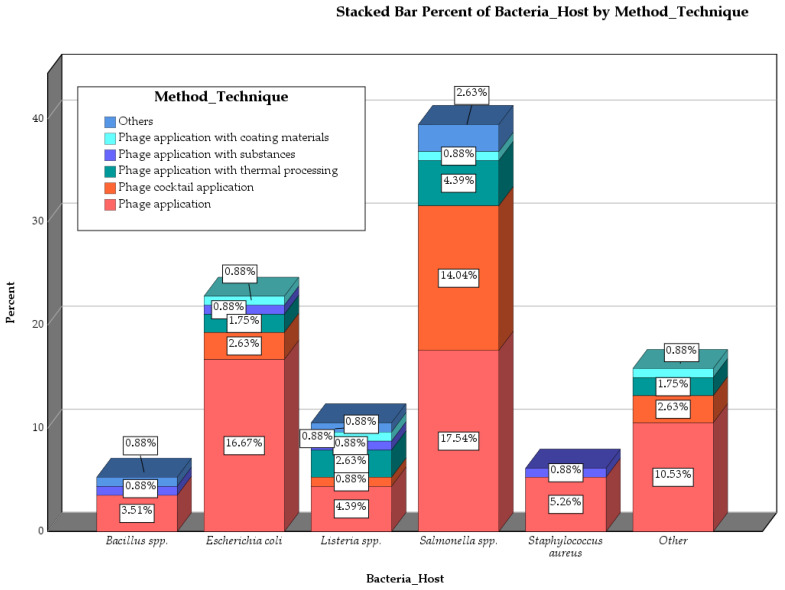
Stacked Bar Chart Showing the Percent Distribution of Bacteria Hosts by Phage Application Technique.

This stacked bar chart (Figure 3) highlights the distribution of phage application techniques (e.g., phage application, phage cocktail, thermal processing, etc.) across different bacterial hosts (*Bacillus* spp., *Listeria* spp., *Salmonella* spp., etc.). The ’Other Bacteria’ category includes *Cronobacter sakazakii*, *Vibrio* spp., *Pseudomonas* spp., *Aeromonas hydrophila*, *Clostridium* spp., *Shigella* spp., and *Enterobacter cloacae* complex (ECC). These species were grouped together due to their relatively low occurrence in the included studies (ranging from 1 to 4 cases), making individual categorization impractical while still maintaining their relevance to the overall analysis. The dominance of specific methods, such as simple phage application, can be observed across various bacterial species, with notable variations in their prevalence depending on the host bacteria.

**Figure 4:**
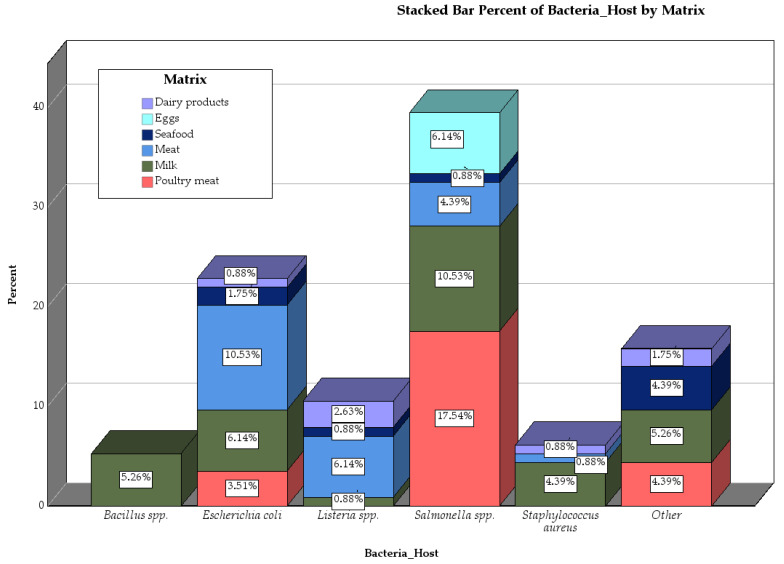
Stacked Bar Chart Representing the Percent Distribution of Bacteria Hosts by Matrix.

The chart (Figure 4) presents the distribution of bacterial hosts across various matrices. *Salmonella* spp. stands out for its prevalence in poultry meat, while *Bacillus* spp. and *Listeria* spp. are more evenly distributed across matrices like dairy products, seafood, and meat. This distribution highlights the varying contamination risks associated with different types of food products, emphasizing the need for targeted bacteriophage interventions depending on the matrix in question.

**Figure 5:**
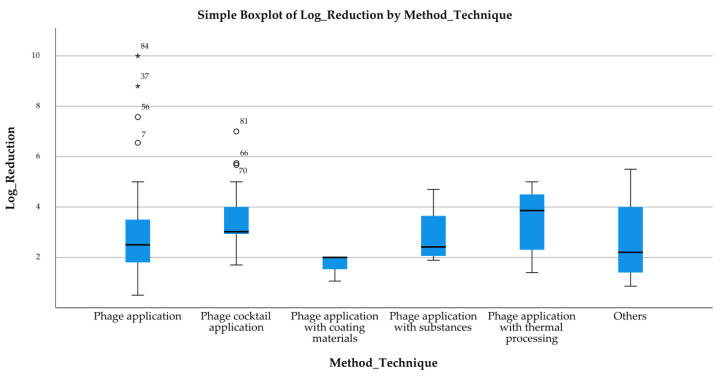
Log Reduction by Method.

**Figure 6:**
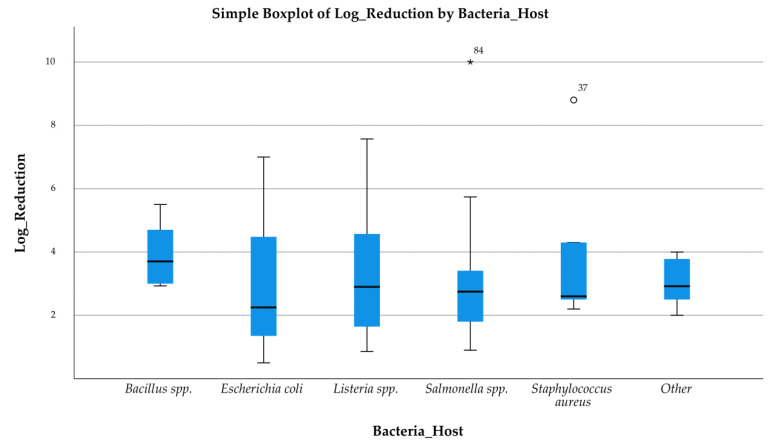
Log Reduction by Bacteria Host.

**Figure 7:**
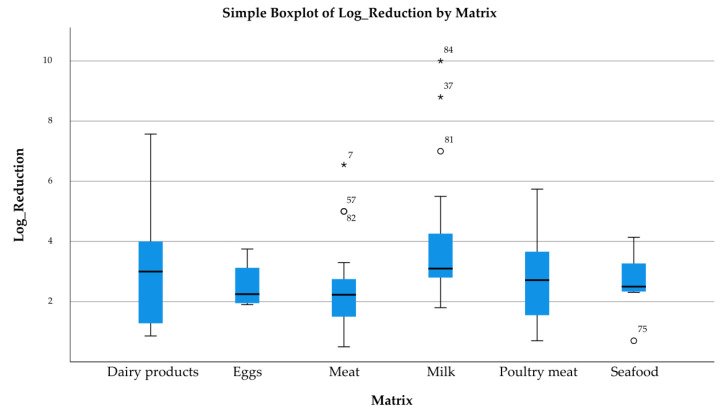
Log Reduction by Matrix.

The boxplots shown in Figure 5, Figure 6 and Figure 7 present the distribution of log reductions of various bacterial hosts and matrices according to different methods used in the analyzed studies. The visual representation highlights the variability and central tendency of log reductions across different experimental categories. The log reductions achieved by different Method Techniques are displayed (Figure 5). The “Phage application” method shows a broader interquartile range (IQR), indicating greater variability in reduction outcomes. Outliers are also noted, suggesting that certain studies produced atypically high reductions. In contrast, “Phage application with thermal processing” yielded consistently higher log reductions with fewer outliers, reflecting a more controlled performance. The 2nd boxplot (Figure 6) focuses on the log reduction outcomes based on Bacteria Hosts. The variability is most pronounced for *Escherichia coli*, where reductions range widely between studies, while *Staphylococcus aureus* and *Bacillus* spp. exhibit more compact distributions, suggesting more consistent log reduction outcomes across studies. Lastly, the 3rd boxplot (Figure 7) presents log reductions categorized by Food Matrices. The distribution for Dairy products is notably broader, suggesting a wide range of reductions across different studies, while Eggs show a narrower range, indicating more consistent results. Some matrices, such as Milk and Meat, also show a considerable spread, with Milk exhibiting several outliers.

These results indicate significant variability in the antimicrobial effectiveness of bacteriophages, depending on the technique, bacterial strain, and food matrix. The presence of outliers and large IQRs in many cases suggests that the performance of bacteriophage applications may be influenced by specific experimental conditions or study designs.

**Figure 8:**
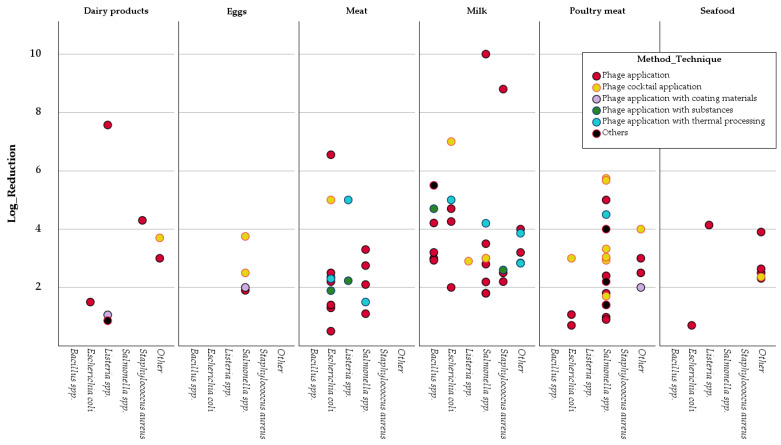
Scatter Plot of Log Reduction by Bacteria Host and Method Technique across Food Matrices.

The scatter plot in Figure 8 illustrates the relationship between the Bacteria Host, Method Technique, and the Log Reduction achieved across various food matrices. This multidimensional visualization allows for a comparative analysis of phage efficacy across different conditions. Each dot in the plot represents a distinct combination of food matrix, bacterial host, and method technique, with the color coding corresponding to the different methods applied. This figure captures six key matrices: Dairy products, Eggs, Meat, Milk, Poultry meat, and Seafood. It shows the variations in log reductions across five main bacterial groups: *Bacillus* spp., *Escherichia coli*, *Listeria* spp., *Salmonella* spp., and *Staphylococcus aureus.*

The scatter plot reveals that *Escherichia coli* and *Salmonella* spp. consistently exhibited the broadest range of log reductions across all matrices. The use of simple Phage application (red dots) is frequent, particularly in Meat, Milk, and Poultry meat matrices, where it often achieves reductions ranging between 1 and 5 logs. Notably, the Phage cocktail application (yellow dots) showed strong results, especially in the Dairy and Poultry meat matrices, where log reductions exceeded 4.0 in some cases. Meanwhile, in matrices such as Eggs and Seafood, phage-based interventions yielded more limited log reductions, typically below 2.0 logs. This suggests that the efficacy of bacteriophage treatment may be contingent upon both the bacterial strain and the food matrix, with varying degrees of success.

Additionally, the figure highlights several outliers, with reductions exceeding 8.0 logs, particularly in the Milk and Dairy products categories when using phage-based thermal processing. Specific phage techniques achieve exceptionally high bacterial reductions in particular contexts.

The statistical analysis (S2 Statistics) revealed a substantial deviation from a normal distribution for the bacterium dose, with a test statistic of 0.192 and a significance value of <0.001. Similarly, the dose of phages showed a statistic of 0.139 and a significance of 0.030, suggesting non-normality, albeit less pronounced. The log reduction also demonstrated non-normality with test statistics of 0.177 and a significance of 0.001.

The Shapiro-Wilk test further supported these findings. For the dose of bacteria, the statistic was 0.887 with a significant value of <0.001, indicating a significant departure from normality. The dose of phages had a statistic of 0.948 with a significance of 0.044, confirming a deviation from normality. Lastly, the log reduction yielded a statistic of 0.890 with a significance of <0.001, again highlighting non-normality.

The Q-Q plots visually complement the statistical tests, showing deviations from the expected normal line for all variables, especially in the tails, further emphasizing the non-normal distribution of the data. Overall, these results indicate that non-parametric tests may be more appropriate for analyzing this dataset.

The results of Spearman’s correlation (S3 Statistics) indicate a statistically significant positive correlation between the dose of bacteria and the dose of phages (ρ = 0.376, *p* = 0.011), suggesting that higher doses of bacteria are associated with higher doses of phages used in the studies. However, no significant correlation was found between the dose of bacteria and log reduction (ρ = −0.100, *p* = 0.515), nor between the dose of phages and log reduction (ρ = −0.043, *p* = 0.777), indicating that there is no clear association between the doses of bacteria or phages and the level of log reduction observed in this dataset. These results suggest that while the dose of bacteria and phages are related, neither appears to directly influence the log reduction outcomes under the conditions studied.

## 4. Discussion

The analysis revealed considerable variability in bacteriophage efficacy across different food matrices. Bacteriophages demonstrated higher efficacy in high-moisture matrices such as poultry and dairy products, with bacterial reductions frequently reaching up to 4 log units. These results suggest that moisture content plays a crucial role in enhancing bacteriophage diffusion, facilitating greater interaction between phages and bacterial cells [92]. The prevalence of pathogens such as *Salmonella* spp. and *Listeria* spp. in these matrices highlights the importance of bacteriophage application as a targeted method for controlling contamination in high-risk food products [93]. However, matrices like seafood and eggs exhibited lower bacteriophage efficacy, with bacterial reductions typically below 2 log units. Despite the high moisture content in seafood, factors such as biofilm formation, surface complexity, and bacterial attachment likely hinder bacteriophage access to bacterial cells [94,95]. For example, biofilms on seafood surfaces can shield bacterial colonies from phage exposure [96]. These findings indicate that while bacteriophages are effective in certain contexts, their application must be tailored to the specific characteristics of each food matrix to optimize bacterial reduction outcomes. This reinforces the necessity for matrix-specific approaches rather than a one-size-fits-all model for phage application.

The treatment method played a significant role in determining the success of bacteriophage applications. Phage cocktails, which utilize a combination of bacteriophages targeting different bacterial strains or mechanisms, consistently produced greater reductions compared to single-phage treatments. This was especially evident in poultry and dairy products, where phage cocktails achieved substantial reductions in bacterial counts, demonstrating their potential for broader-spectrum biocontrol in the food industry. These results align with studies [97,98], which also found enhanced efficacy of phage cocktails in various matrices compared to single-phage treatments. The multi-strain targeting capacity of phage cocktails not only improves efficacy but also helps mitigate bacterial resistance [99]. Single-phage treatments, on the other hand, exhibited greater variability in outcomes. While some studies reported significant bacterial reductions, others showed minimal effects, suggesting that single-phage treatments may be more susceptible to variations in experimental conditions. These findings highlight the importance of using combination treatments or phage cocktails to ensure more consistent and reliable bacterial reductions across different food matrices and bacterial strains. Moreover, thermal processing combined with phage application emerged as a particularly effective strategy, as indicated by the scatter plots comparing method and log reduction. Treatments that involved phage cocktails or thermal interventions consistently resulted in more significant reductions across bacterial hosts and matrices. By integrating phages with other antimicrobial interventions, these methods create a multifaceted approach to bacterial control that can overcome the limitations of single-phage applications. Similar results were observed in studies showing that the combination of phages with heat treatment disrupts bacterial cell integrity [100], thereby improving phage activity and enhancing bacterial reduction [63]. The scatter plots highlight those treatments incorporating thermal processing and phage cocktails achieved consistently greater reductions compared to other methods.

One of the more surprising findings was the absence of a clear dose-response relationship between bacteriophage dose and log reduction. Although a statistically significant correlation was found between the dose of bacteria and the dose of phages (ρ = 0.376, *p* = 0.011), neither was significantly correlated with the final bacterial reduction observed. This lack of a dose-response relationship challenges the traditional assumption that increasing bacteriophage concentrations will inherently lead to greater bacterial reductions. Several factors may explain this unexpected result. First, the characteristics of the food matrix itself could play a significant role in limiting bacteriophage effectiveness, regardless of dose. For example, the presence of biofilms or matrix-specific barriers may prevent phages from reaching bacterial cells in sufficient quantities to produce meaningful reductions, even when higher doses are applied. Additionally, the variability in bacterial strain virulence and phage-host interactions likely contribute to the inconsistency in results. It is also possible that certain bacterial strains are more resistant to phage treatment, particularly in certain environmental conditions, further complicating the relationship between dose and bacterial reduction. These findings suggest that optimizing bacteriophage treatments requires more than simply adjusting the dose. Instead, a holistic approach that considers matrix-specific factors, bacterial characteristics, and treatment combinations may be necessary to achieve reliable and substantial bacterial reductions in food products.

The findings of this review offer valuable insights into the practical applications of bacteriophages in food safety, particularly in the disinfection of animal-derived products. The demonstrated success of bacteriophage treatments in poultry and dairy products suggests that phages could serve as effective alternatives or supplements to chemical disinfectants in these sectors. With reductions of up to 4 log units, bacteriophages hold significant promise for mitigating the risk of foodborne pathogens such as *Salmonella* spp., *Listeria* spp., and *Escherichia coli*. However, the variability in efficacy across food matrices suggests that bacteriophage applications must be tailored to specific environments. The limited success of phage treatments in matrices like seafood and eggs highlights the importance of developing targeted application protocols that account for the unique characteristics of each food product. Additionally, the absence of a dose-response relationship emphasizes the need for further research into optimizing bacteriophage treatments based on factors beyond just dose, such as bacterial resistance mechanisms and matrix permeability. The heterogeneity of the included studies, in terms of bacterial strains, phage types, and application methods, likely contributed to the variability in results. Many of the studies were conducted under controlled laboratory conditions, which may not fully reflect the challenges of applying bacteriophage treatments in commercial food processing environments. Future research should focus on standardizing bacteriophage application protocols to ensure consistency across studies and to better assess the scalability of these treatments in real-world food production settings. Investigating the environmental factors that influence phage efficacy, such as temperature, pH, and biofilm formation, would also provide valuable insights into optimizing treatments for different food matrices. Additionally, further research into the long-term effects of bacteriophage use in food safety is essential, particularly concerning the potential development of bacterial resistance to phages. One of the key mechanisms by which bacteria develop resistance to bacteriophages is through the CRISPR-Cas system, which enables them to acquire adaptive immunity against phages by incorporating fragments of viral DNA into their genome [101]. Developing phage cocktails, engineered phages, and rotating different phage strains over time have been proposed as potential solutions to mitigate the emergence of resistance and sustain the efficacy of phage-based disinfection treatments.

## 5. Conclusions

This systematic review highlights the significant potential of bacteriophages in reducing bacterial contamination in animal-derived food products, particularly in high-moisture environments like poultry and dairy. However, the results demonstrate that phage efficacy varies considerably depending on the type of food matrix and the treatment method used. The findings emphasize the importance of tailoring bacteriophage applications to specific food matrices and bacterial strains, rather than relying on a one-size-fits-all solution. Phage cocktails and their combination with thermal treatments showed particularly strong results, indicating that multi-faceted approaches hold great promise for enhancing bacterial control. Nevertheless, the absence of a clear dose-response relationship across studies suggests that optimizing bacteriophage treatments will require a more targeted and nuanced approach. Environmental conditions, bacterial resistance mechanisms, and phage-host dynamics all play critical roles in determining the success of these treatments. Future research should focus on refining application protocols, ensuring consistent long-term efficacy, and addressing concerns about bacterial resistance. Bacteriophages, if properly harnessed, could play a transformative role in modern food safety strategies, complementing or even replacing conventional antimicrobial methods. However, their integration into food safety practices will depend on a deeper understanding of the variables affecting their performance and the development of standardized, adaptable application methods.

## Supplementary Materials

The following supporting information can be downloaded at: https://www.mdpi.com/article/10.3390/pathogens14030291/s1, Table S1: Overview of Articles Analyzed: Parameters and Study Characteristics; S2 Statistics: Kolmogorov-Smirnov Test, Normality, QQ plots; S3 Statistics: Spearman Correlation

## Abbreviations

The following abbreviations are used in this manuscript:

ECDC	European Centre for Disease Prevention and Control
CDC	Centre for Disease Prevention and Control
TASF	Terrestrial Food of Animal Origin
ISO	International Organization for Standardization
HACCP	Hazard Analysis & Critical Control Point
FDA	Food and Drug Administration
PRISMA	Preferred Reporting Items for Systematic reviews and Meta-Analyses
IQR	Interquartile Range
