# Supplementary material for: Can Bacteriophages Be Effectively Utilized for Disinfection in Animal-Derived Food Products? A Systematic Review
Source: Pathogens. 2025 Mar 16;14(3):291. doi: 10.3390/pathogens14030291 (PMC11944998; doi:10.3390/pathogens14030291)
Supplement: Supplementary file 1 [file pathogens-14-00291-s001.zip › pathogens-3534739-Table S1.pdf]

Supplementary Material

Table 1. "Overview of Articles Analyzed: Parameters and Study Characteristics"

Title	Country	Host	Dose of Bacteria	Bacteriophage	Dose of bacteriophages	Host range study (Infection efficacy)	Stability of phage in temperature & pH	Adsorption rate (adsorption efficacy (%), adsorption time)	Treatment time	Matrix	Method	Reduction log
**A broad-spectrum phage controls multidrug-resistant Salmonella in liquid eggs**	China	Salmonella spp.	10^5 CFU/mL	D1-2	10^8 or 10^9 PFU/mL	Broad host spectrum against Salmonella strains, including multidrug-resistant Salmonella Typhimurium και Salmonella Enteritidis	Stable at 30-50°C, pH 4-12	64.12% at 40 min	1, 3, 6, 12, and 24 hours at varying temperatures	Eggs	Phage application	Significant reduction
**A Genomic Analysis of the Bacillus Bacteriophage Kirovirus kirovense Kirov and Its Ability to Preserve Milk**	Russia	Bacillus spp.	10^5 CFU/mL	Kirovirus kirovense Kirov	10^4 PFU/mL	Infects 12 out of 42 Bacillus strains, with significant efficacy against Bacillus cereus	Stable at 20-50°C, pH 5-11	N/A	4 hours	Milk	Phage application	~ 3 log
**A novel Bacillus cereus bacteriophage DLn1 and its endolysin as biocontrol agents against Bacillus cereus in milk**	China	Bacillus spp.	10^4 CFU/mL	DLn1	different PFU/CFU ratios of 10^2 and 10^3 (N/A)	Narrow but specific host range to Bacillus cereus strains	Stable at 4-55°C, pH 4-10	High burst size (618 PFU/cell), 15 min latent period	0, 3, 6, 12, and 24 h	Milk	Phage application & Phage application with substances	**Phage DLn1:**<br>After 3 hours: Reduction of 0.9 log10 CFU/mL (PFU/CFU ratio 100) and 2.3 log10 CFU/mL (PFU/CFU ratio 1000).<br>After 6 hours: Significant reduction, minimum bacterial counts reached.<br>After 12 hours: Reduction of approximately 3.2 log10 CFU/mL.<br>After 24 hours: Bacterial counts nearly the same as the control group<br>**Endolysin:** Reduction of 0.8 log10 CFU/mL after 3 hours, with a continuous inhibitory effect, reaching 4.7 log10 CFU/mL reduction after 24 hours.
**A Polyvalent Broad-Spectrum Escherichia Phage Tequatrovirus EP01 Capable of Controlling Salmonella and Escherichia coli Contamination in Foods**	China	Salmonella spp. & Escherichia coli	10^8 CFU/mL	Tequatrovirus EP01	MOIs of 10^-3 and 1	Broad host range against multiple strains of Salmonella και Escherichia coli	Stable at 30-80°C, pH 4-10	Short latent period (10 min), large burst size (80 PFU/cell)	2, 4, 6, 24 h	Milk & Meat	Phage application	**Milk-Salmonella** : 3.5 log10 CFU/mL at 4°C and 2.5 log10 CFU/mL at 28°C after 24 hours.<br>**Milk-Escherichia.coli :** 3.3 log10 CFU/mL at 4°C and 28°C after 24 hours,<br>4.26 log10 CFU/mL at 4°C and 28°C after 24 hours<br>**Meat Salmonella :** 3.3 log10 CFU/cm^2 at 4°C and 2.5 log10 CFU/cm^2 at 28°C after 24 hours<br>**Meat-Escherichia coli :** 5.53 log10 CFU/cm^2 , 2.18 log10 CFU/cm^2 & 6.55 log10 CFU/cm^2 at 4°C and 28°C after 24 hours.

**Anti-Salmonella polyvinyl alcohol coating containing a virulent phage PBSE191 and its application on chicken eggshell**	Korea	Salmonella spp.	2.4 × 10^8 CFU/mL	PBSE191	4.0 × 10^9 PFU/mL	Active against a broad range of Salmonella isolates, including six serotypes	Stable in a wide range of temperatures (-18°C to 80°C) and pH (1-9), with optimal stability at pH 4-9	99.85% adsorption to bacterial cell surface within 25 minutes	24h	Eggs	Phage application with coating materials	1 log CFU immediately after coating,<br>2 log CFU within 24 h on chicken eggshells
**Application of a novel lytic phage vB_EcoM_SQ17 for the biocontrol of Enterohemorrhagic Escherichia coli O157:H7 and Enterotoxigenic E. coli in food matrices**	China	Escherichia coli	10^4 CFU/mL	vB_EcoM_SQ 17	10^8 PFU/ml	Active against EHEC O157<br>, ETEC, and other E. coli strains	Stable between pH 4-12 and at 30°C-60°C for 60 minutes	10 min latent period, burst size of 71 PFU/infected cell	24h	Milk & Meat	Phage application	**Milk:** ~4.7 log CFU/mL to below the detection limit after 4 h &<br>~4.5 log CFU/ml to below the detection limit after 8 h,<br>12 and 24 h : e ≥3.43 and 1.01 log CFU/ml<br>**Meat:** Reduction of 2.35 log CFU/piece (p < 0.05) at 4°C after 12 hours.,<br>0.70 log CFU/piece after 4 hours
**Application of a novel phage LPCS28 for biological control of Cronobacter sakazakii in milk and reconstituted powdered infant formula**	China	Other	10^3 CFU/mL	LPCS28	10^6 PFU/mL or 10^7 PFU/mL, 9×10^5 PFU/mL or 10^7 PFU/mL	Active against 92.5% (25/27) of different species and genera strains tested	Stable between pH 4-10 and 60°C for 1 hour	Latent period of 20 minutes, burst size of 81 PFU/cell	12 h (bacterial enumeration on LA plates after 0, 2, 4, 6, 9, and 12 h of incubation)	Milk	Phage application with thermal processing	**Liquid milk:** the inhibitory rates were 99.99% (MOI = 10000, reduced by 3.86 log10 CFU/mL) and 99.97% (MOI = 1000, reduced by 3.59 log10 CFU/mL) at 6 h. When the incubation time reached 9 h, the bacterial concentration in both liquid milk and RPIF samples decreased to below the limit of detection (<10 CFU/mL) for both the MOI tested, and no recovered bacterial cell was observed after 12 h of incubation<br>**RPIF:** 2.83 log10 CFU/mL (6 hours), MOI of 1000 was 99.72% (reduced by 2.56 log10 CFU/mL) for liquid milk
**Application of a novel phage vB_SalS-LPSTLL for the biological control of Salmonella in foods**	China	Salmonella spp.	10^3 CFU/mL	vB_SalS-LPSTLL	10^6 PFU/mL or 10^7 PFU/mL	Active against 11 serotypes of Salmonella	Stable between pH 3-12 at 60°C fot 60 min	Latent period of 10 minutes, burst size of 71 PFU/cell	12 h (enumeration on 1,3,6,12 h)	Milk & Poultry Meat	Phage application	**Milk:** 2.8 log CFU/mL at 25°C και 0.9 log CFU/mL at 4°C<br>**Raw chicken:** 0.71 log CFU/sample at MOI 1000 και 0.98 log CFU/sample at MOI 10000 at 25°C
**Application of a Phage Cocktail for Control of Salmonella in Foods and Reducing Biofilms**	China	Salmonella spp.	10^3 CFU/mL	LPSTLL, LPST94 and LPST153	"MOI of 10^3 - 10^7 PFU/mL, MOI of 10^3 - 10^6 log10 PFU/cm2 or MOI of 10^4 - 10^7 PFU/cm2	Active against Salmonella serovars (Typhimurium, Enteritidis, and others)	Stable at 4°C και 25°C	N/A	3-24 h	Milk	Phage cocktail application	**Milk:** was reduced below detectable limit (<1 CFU/100 μL) after 3 h and 6 h<br>**Chicken breast:** no viable count could be recovered by direct plating after 3 h incubation, the Salmonella counts were eliminated completely after 3 h and 6 h upon application of phage cocktail at an MOI of 10000 and 1000

**Application of bacteriophages EP75 and EP335 efficiently reduces viable cell counts of Escherichia coli O157 on beef and vegetables**	Netherlands, Switzerland	Escherichia coli	10^5 CFU/cm^2	EP75 & EP335	2 × 10^7 or 1 × 10^8 PFU/cm^2	Active against 83.0% (73/88) and 86.4% (76/88) of E. coli O157 isolates tested	Stable at 4°C	N/A	30 min -24 h	Meat	Phage application	2 × 10^7 PFU/cm^2: 0.8–1.1 log10 CFU/cm^2 reduction<br>1 × 10^8 PFU/cm^2: 0.9–1.3 log10 CFU/cm^2 reduction
**Application of novel lytic bacteriophages to control Vibrio parahaemolyticus load in seafood**	India	Other	6.9 × 10^6 CFU/mL	VpPH	2 × 10^10 PFU/mL	Broad host range (62.2%) among V. parahaemolyticus isolates	N/A	Maximum lytic activity within 4 h	0, 4, 8, and 12 h	Seafood	Phage application	2.5 log10 CFU/g after 12 hours
**Bacteriophage ϕIBB-PF7A loaded on sodium alginate-based films to prevent microbial meat spoilage**	Portugal	Other	~10^6 CFU/cm^2	ϕIBB-PF7A	10^8 PFU/mL	Broad-host range	Stable under refrigerated conditions (4°C) for up to 8 weeks	N/A	0-7 days	Poultry meat	Phage application with with coating materials	2 log CFU reduction in P. fluorescens within the first 2 days<br>1 log CFU reduction maintained up to 5 days
**Bio-control of O157:H7, and colistin-resistant MCR-1-positive Escherichia coli using a new designed broad host range phage cocktail**	China	Escherichia coli (colistin-resistant **MCR** strain)	10^4 CFU/mL	EK010, O18-011, and vB_EcoM_005	10^12 PFU/mL or 10^8 PFU/cm^2	Broad host range	Stable at -20°C to 37°C; inactive at 70°C, stable in pH 7-9	Maximum efficacy achieved within 6 hours	0, 1, 3, 12 h, 1, 3, and 7 days	Poultry meat	Phage cocktail application	**Raw Chicken Meat:** 3 log CFU reduction within the first hour,<br>Below detection limit after 6 hours<br>No viable E. coli recovered until day 7
**Biocontrol of Salmonella Typhimurium in milk, lettuce, raw pork meat and ready-to-eat steamed-chicken breast by using a novel bacteriophage with broad host range**	China, Japan, Egypt	Salmonella spp.	10^4 CFU/mL	PS3-1	MOI 10^4 - 10^8 PFU/mL	Broad host range (60% of tested Salmonella strains)	Stable between 4-60°C and pH 3.0-11.0	Maximum adsorption efficiency (93.3%) within 4 minutes	0, 2, 4, 6, 12, 24 and 48 h	Milk, Meat & Poultry Meat	Phage application	**Milk:** At 7°C: 1.34 log reduction after 2h, stable up to 48h,<br>At 25°C: 2.08 log reduction after 4h, some regrowth observed after 24h<br>At 37°C: 2.19 log reduction after 2h, some regrowth observed after 24h<br>**Raw Pork Meat:**<br>At 7°C: 1.38 log reduction after 2h, stable up to 48h<br>At 25°C: 2.75 log reduction after 4h, stable up to 24h<br>At 37°C: 2.60 log reduction after 2h, stable up to 24h<br>**Ready-to-Eat Steamed-Chicken Breast:**<br>At 7°C: 1.68 log reduction after 2h, stable up to 48h<br>At 25°C: 1.50 log reduction after 4h, stable up to 48h<br>At 37°C: 1.80 log reduction after 2h, stable up to 24h

**Biological Characterization of Pseudomonas fluorescens Phage Pf17397_F_PD1 and Its Application in Food Preservation**	China	Other	10^3 CFU/mL	Pf17397_F_PD 1 (PD1)	MOIs of 10^2 and 10^3 - 10^5 and 10^6 PFU/mL	Specific for P. fluorescens ATCC 17397	Stable between 30-70°C and pH 4-11	Maximum adsorption within 18 minutes, burst size of 1.47 × 10^2 PFU per infected cell	0 h,<br>6 h, 12 h, 24 h, 48 h, and 72 h	Milk & Seafood	Phage application	**Milk:** At 4°C: 2.41 log CFU/mL reduction (MOI 100) and 2.56 log CFU/mL reduction (MOI 1,000) after 72h<br>At 25°C: 2.71 log CFU/mL reduction (MOI 100) and 2.84 log CFU/mL reduction (MOI 1,000) after 48h<br>**Grass Carp:** At 4°C: 0.35 log CFU/g reduction (MOI 100) and 0.96 log CFU/g reduction (MOI 1,000) after 48h, At 25°C: 1.28 log CFU/g reduction (MOI 100) and 2.64 log CFU/g reduction (MOI 1,000) after 24h
**Broad host range phage vB-LmoM-SH3-3 reduces the risk of Listeria contamination in two types of ready-to-eat food**	China	Listeria spp.	10^5 CFU/g	vB-LmoM-SH3-3	10^8 PFU/g	95% (43/45) of Listeria isolates lysed	Stable from 4°C to 60°C	Short latent period (~10 minutes), high burst size (~714.29 PFU/cell)	0, 4, 16, 24, 48 and 72 hours	Seafood	Phage application	**Salmon Meat:**<br>2.67 log CFU reduction after 24 hours at 4°C<br>4.14 log CFU reduction after 48 hours at 4°C<br>Below detection limit after 72 hours at 4°C
**Broad-Spectrum Salmonella Phages PSE-D1 and PST-H1 Controls Salmonella in Foods**	China	Salmonella spp.	1.0 × 10^8 CFU/mL (Eggs), 4.0 × 10^5 CFU/mL (Meat), 10^5 CFU/mL (Milk)	Salmonella phage PSE-D1 and Salmonella phage PST-H1	MOIs 10 and 10^2	Effective against multiple Salmonella, E. coli, and K. pneumoniae strains	Stable at 4-60°C and pH 3-11	PSE-D1: 10 min latent period, PST-H1: 20 min latent period	**Eggshells:** and sausages 2, 4, 6, 24, and 48 hours, **Milk:** 2, 4, 6, and 24 hours	Eggs, Meat & Milk	Phage application	**Eggshells: At 4°C:**<br>PSE-D1: 1.5 log CFU/sample reduction (MOI 100), 1.1 log CFU/sample reduction (MOI 10) after 48h<br>PST-H1: 1.9 log CFU/sample reduction (MOI 100), 1.4 log CFU/sample reduction (MOI 10) after 48h<br>**At 28°C:**<br>**PSE-D1**: 1.0 log CFU/sample reduction (MOI 100), 0.9 log CFU/sample reduction (MOI 10) after 48h<br>**PST-H1**: 1.4 log CFU/sample reduction (MOI 100 and MOI 10) after 48h<br>**Sausages: At 4°C:**<br>**PSE-D1**: 1.1 log CFU/cm^2 reduction (MOI 100 and MOI 10) after 48h<br>**PST-H1**: 0.9 log CFU/cm^2 reduction (MOI 100), 1.0 log CFU/cm^2 reduction (MOI 10) after 48h<br>At 28°C:<br>**PSE-D1**: 0.6 log CFU/cm^2 reduction (MOI 100) after 48h<br>**PST-H1**: 0.8 log CFU/cm^2 reduction (MOI 100), 0.7 log CFU/cm^2 reduction (MOI 10) after 48h<br>**Milk: At 4°C:**<br>**PSE-D1:** 1.5 log CFU/mL reduction (MOI 100), 1.2 log CFU/mL reduction (MOI 10) after 24h<br>**At 28°C:**<br>**PSE-D1**: 1.0 log CFU/mL reduction (MOI 100), 0.7 log CFU/mL reduction (MOI 10) after 24hfter<br>24 h incubation
**CAM-21, a novel lytic phage with high specificity towards Escherichia coli O157:H7 in food products**	USA	Escherichia coli	10^5 CFU/mL, 10^5 CFU/g	CAM-21	MOIs 10^3 and 10^4 - 10^9 PFU/mL	Effective against multiple E. coli serotypes	Stable under refrigeration (4°C)	~ 49% adsorbed within 1 minute, 88% adsorbed within 15 minutes	0, 3, 6, 9, 12, and 24 hours	Milk & Meat	Phage application	**Milk:** At 4°C:<br>MOI 1000: 1.4 log CFU/mL reduction after 24 hours<br>MOI 10,000: 2.0 log CFU/mL reduction after 24 hours<br>**Ground Beef:** At 4°C:<br>MOI 1000: 1.4 log CFU/g reduction after 24 hours<br>MOI 10,000: 1.3 log CFU/g reduction after 24 hours

**Characterization and application of a lytic jumbo phage ZPAH34 against multidrug-resistant Aeromonas hydrophila**	China	Other	10^6 CFU/mL	ZPAH34	MOIs 10 and 10^2 - 10^7 PFU/mL, 10^8 PFU/mL	Specific to Aeromonas hydrophila ZYAH75	Stable between 30-50°C and pH 4-11	Approximately 78.5% adsorbed within 5 minutes, 94.45% adsorbed within 15 minutes	0, 1, 3, and 6 hours	Seafood	Phage application	**Fish Fillets (Grass Carp):**<br>**At 4°C:**<br>MOI 10: 1.48 log CFU/sample reduction after 6 hours<br>MOI 100: 1.49 log CFU/sample reduction after 6 hours<br>**At 25°C:**<br>MOI 10: 1.84 log CFU/sample reduction after 1 hour, 2.0 log CFU/sample reduction after 3 hours, 1.01 log CFU/sample reduction after 6 hours<br>MOI 100: 1.71 log CFU/sample reduction after 1 hour, 2.31 log CFU/sample reduction after 3 hours, 1.0 log CFU/sample reduction after 6 hours
**Characterization and Application of a Lytic Phage D10 against Multidrug-Resistant Salmonella**	China	**(multidrug-resistant)** Salmonella spp.	10^5 CFU/mL	D10, combined with myovirus D1-2 and podovirus Pu20	MOIs of 10^3 and 10^4 - 10^9 PFU/mL	Effective against multiple MDR Salmonella serovars, lysed 23 out of 26 tested Salmonella strains	Stable between pH 4-12, moderate thermal tolerance	Approximately 47.4% adsorbed within 20 minutes	0, 1, 3, 6, 12, and 24 hours	Eggs	Phage cocktail application	**Liquid Egg Whites: At 4°C**:<br>MOI 1000: Significant reduction in bacterial count, continuous reduction observed throughout the test period<br>MOI 10,000: Effective in reducing viable bacteria at all designated time points<br>**At 25°C:**<br>MOI 1000: Significant decreases in bacterial counts observed at 12 and 24 hours<br>MOI 10,000: Effective in reducing viable bacteria at all designated time points<br>**Liquid Egg Yolks: At 4°C:**<br>MOI 1000 and 10,000: Successfully diminished the bacterial burden throughout the test period<br>**At 25°C:**<br>MOI 1000 and 10,000: Successfully diminished the bacterial burden throughout the test period
**Characterization and Food Application of the Novel Lytic Phage BECP10: Specifically Recognizes the O-polysaccharide of Escherichia coli O157:H7**	Korea	Escherichia coli	10^6 CFU/mL	BECP10	10^8 PFU/mL	Specific to E. coli O157 strains, did not infect non-O157 strains or other Gram-negative/positive bacteria	Stable between 25-45°C, active at pH 4-9	Approximately 70% adsorbed within 3 minutes, adsorption completed within 15 minutes	0, 24, 48, 72, 96 & 120 hours	Meat	Phage application	1.8 log reduction within 48 hours, 2.2 log reduction within 96 hours, Below detection limit after 120 hours
**Characterization of Clostridium perfringens bacteriophages and their application in chicken meat and milk**	Japan	Other	10^7 CFU/mL (Poultry Meat), 10^6 CFU/mL (Milk)	CPQ1	MOI of 10 - 5 × 10^7 PFU/mL (Poultry Meat), MOI of 10 (Milk)	Specific to Clostridium perfringens strains	Stable between 4-9 pH and up to 60°C	Latent period of 50 minutes, burst size of 6510 ± 859 PFU/bacterial cell	0, 2, 4, 6, and 24 hours	Poultry meat & Milk	Phage application	**Chicken Meat:** 1.2 log reduction at 24°C within 6 hours, 2.5 log reduction at 37°C within 2 hours<br>**Milk:** 0.4 log at 24 ◦C, after 24 h -2 log and more than 4 log at 24 ◦C and 37 ◦C, respectively

**Characterization of G-type Clostridium perfringens bacteriophages and their disinfection effect on chicken meat**	China	Other	10^7 CFU/mL	ZWPH-P21 (P21)	1 x 10^6 PFU/mL, 1 x 10^7 PFU/mL, 1 x 10^8 PFU/mL	Specific to Clostridium perfringens type G	Stable at 30-50°C, pH 4-11	Highest titer at MOI of 0.1, with latent period of 20 minutes	0, 2, 12, 24, 36 and 48 hours	Poultry meat	Phage application	**Chicken Meat:**<br>At 25°C (room temperature):<br>1 × 10^6 PFU/mL: Reduced to 10^4 CFU/mL within 12 hours, controlled at this level up to 36 hours<br>1 × 10^7 PFU/mL: Reduced to <10^3 CFU/mL within 12 hours, began to rise after 24 hours<br>1 × 10^8 PFU/mL: Reduced to <10^3 CFU/mL within 12 hours, began to rise after 24 hours
**Characterization of KMSP1, a newly isolated virulent bacteriophage infecting Staphylococcus aureus, and its application to dairy products**	Korea	Staphylococcus aureus	10^4 CFU/mL (Milk), 10^4 CFU/cm 2 (Dairy products)	KMSP1	MOIs of 10^3 or 10^4	Broad spectrum of S. aureus strains	Stable between 4-55°C, pH 5-11	95% adsorbed within 15 minutes, >99% within 25 minutes	0.5, 3, 6, 12, and 24 hours	Milk & Dairy products	Phage application	**Milk:** at MOI 10^3 - 5.3 log CFU/mL after 24 h at 25 ◦C, MOI 10^4- (1.0 log CFU/mL) after 12 h, MOI 10^4: Significant reduction of log 8.8 CFU/mL after 24 hours<br>**Cheese:** MOI of 10^3<br>and 10^4-0.6 log<br>and 1.4 log CFU/cm2, respectively, after 30 min at 25 ◦C, MOI 10^4: Significant reduction of log 4.3 CFU/cm² after 24 hours
**Characterization of Phage vB_SalM_SPJ41 and the Reduction of Risk of Antibiotic-Resistant Salmonella enterica Contamination in Two Ready-to-Eat Foods**	China	Salmonella spp.	10^6 CFU/mL	SPJ41	2 × 10^8 PFU/g	Broad spectrum against multiple Salmonella serovars (lysed 11 strains of S. enterica across 5 serovars)	Active at temperatures <80°C; stable in pH range 3-11	Approximately 70% adsorption within 3 minutes, completed within 15 minutes	0, 3, 6, 9, 12, and 24 hours	Seafood	Phage application	**Salmon:**<br>At 4°C: 2.2 log CFU/g reduction for S. Enteritidis at 24 hours; 0.7 log CFU/g reduction for MDR S. Derby at 3 hours, 1.1 log CFU/g reduction at 24 hours<br>At 15°C: 3.9 log CFU/g reduction for S. Enteritidis at 24 hours; 2.1 log CFU/g reduction for MDR S. Derby at 24 hours
**Characterization of Salmonella spp.-specific bacteriophages and their biocontrol application in chicken breast meat**	Korea	Salmonella spp.	9.5 × 10^5 CFU/mL	CAU-SEP-1, CAU-SEP-2, CAU-SEP-3, CAU-SEP-4 (cocktail)	MOI 10^4, 10^5 and 10^6 - 10^8,10^9, and 10^10 PFU/mL	Broad spectrum against multiple Salmonella serovars,lysed 96% of 25 Salmonella strains	Stable at 60-65°C, pH 5-11	50% adsorbed within 1-6 min, nearly 100% within 18-40 min	1, 2, 3, 4, 5, 6, and 7 days	Poultry meat	Phage cocktail application	MOI 10^4: Reduction of 0.52 to 1.51 log CFU/cm² over 7 days<br>MOI 10^5: Reduction of 0.8 to 1.64 log CFU/cm² over 7 days<br>MOI 10^6: Reduction of 1.36 to 2.93 log CFU/cm² over 7 days

**Characterization of Salmonella Thompson-specific bacteriophages and their preventive application against Salmonella Thompson biofilm on eggshell as a promising antimicrobial agent in the food industry**	Korea	Salmonella spp.	10^4 CFU/mL	CAU-STP-1 and CAU-STP-2 (cocktail)	MOI of 10^2, 10^3, and 10^4 - 10^7, 10^8, or 10^9 PFU/mL	Specific to Salmonella Thompson	Stable at 60-65°C, pH 4-11	60% absorbed within 5 minutes for CAU-STP-2	1, 2, 3, 4, 5, 6, and 7 days	Eggs	Phage cocktail application	(10◦C)-MOI of 10^3: 0.03, 0.07, 0.10, 0.11, 0.11, 0.05, and 0.26 log CFU/cm2, MOI 10^4: 0.13, 0.12, 0.13, 0.14, 0.19, 0.45, and 0.96 log CFU/cm2, MOI 10^5: 0.18, 0.19, 0.22, 0.45, 0.72, 1.42, and 2.50 log CFU/cm2-1, 2, 3, 4, 5, 6, and 7, respectively. (20 ◦C)- MOI of 10^3: 0.11, 0.14, 0.20, 0.09, 0.20, 0.06, and 0.11 log CFU/cm2, MOI 10^4: 0.17, 0.17, 0.26, 0.17, 0.28, 0.22, and 0.24 log CFU/cm2, MOI 10^5: 0.21, 0.24, 0.35, 0.31, 0.38, 0.29, and 0.33 log CFU/cm2
**Characterization of the lytic phage MSP1 for the inhibition of multidrug-resistant Salmonella enterica serovars Thompson and its biofilm**	Korea	MDR Salmonella spp.	10^4 CFU/mL (Poultry meat) , 10^5 CFU/mL (Milk)	MSP1	10^8–10^9 PFU/mL	Broad spectrum against multiple Salmonella serovars	Stable at 25-55°C, pH 4-11	50% adsorption within 1-6 min, nearly 100% within 18-40 min	3, 6, 9, and 12 hours	Poultry meat & Milk	Phage application	**Chicken:** at 4 ◦C, 10^8 PFU/mL - 1.5 log reduction after 3 h, 10^9 PFU/mL - below the detection limit after 3 h, At 20°C: 10^9 PFU/mL: Reduction to below detection limit after 3 hours **Milk:** at 4 ◦C, 3.5 log CFU/mL, 10^8 PFU/mL, not detected at 10^9 PFU/mL, At 20°C: 10^8 PFU/mL: Complete loss of viability after 6 hours 10^9 PFU/mL: Complete loss of viability after 3 hours
**Characterization of two novel lytic bacteriophages for reducing biofilms of zoonotic multidrug-resistant Staphylococcus aureus and controlling their growth in milk**	Egypt	**MDR** Staphylococcus aureus	10^8 CFU/mL	vB_SauM_ME 18 & vB_SauM_ME 126	MOIs of 1 and 10 - 10^8 PFU/ml	Specific to MDR S. aureus isolates	Stable at 4-37°C, pH 4-11	~95% within 15-25 minutes	2 up to 6 hours	Milk	Phage application	**UHT Milk:** At 25°C: vB_SauM_ME126 (MOI 10): Complete eradication of S. aureus after 6 hours vB_SauM_ME18 (MOI 10): Complete eradication of S. aureus after 6 hours At 37°C: vB_SauM_ME126 (MOI 10): Complete eradication of S. aureus after 4-6 hours vB_SauM_ME18 (MOI 10): 87.2% reduction in CFU/mL after 6 hours

**Combine thermal processing with polyvalent phage LPEK22 to prevent the Escherichia coli and Salmonella enterica contamination in food**	China	Escherichia coli & Salmonella	10^4 CFU/cm 2 (E. coli- Meat), 10^4 CFU/cm 2 (Salmonella- Meat), 10^4 CFU/mL (E. coli- Milk), 10^4 CFU/mL (Salmonella- Milk)	LPEK22	MOIs 10^2 or 10^3	Effective against E. coli and Salmonella enterica	Stable at 60-65°C, retains 50% activity at 70°C; stable at pH 4-12	97% adsorption within 10 minutes	0, 1, 3, 6, 9, and 12 hours	Meat & Milk	Phage application with thermal processing	**Escherichia coli - Meat :**<br>MOI 100 at 4°C :<br>Reduction: 1.0 log10 CFU/cm²<br>after 12 hours<br>MOI 1000 at 4°C:<br>Reduction: 1.4 log10 CFU/cm²<br>after 12 hours<br>MOI 100 at 25°C:<br>Reduction: 1.7 log10 CFU/cm²<br>after 12 hours<br>MOI 1000 at 25°C:<br>Reduction: 2.3 log10 CFU/cm²<br>after 12 hours<br>**Salmonella-Meat :** MOI 100 at 4°C:<br>Reduction: 0.9 log10 CFU/cm²<br>after 12 hours<br>MOI 1000 at 4°C:<br>Reduction: 1.1 log10 CFU/cm²<br>after 12 hours<br>MOI 100 at 25°C:<br>Reduction: 1.4 log10 CFU/cm²<br>after 12 hours<br>MOI 1000 at 25°C:<br>Reduction: 1.5 log10 CFU/cm²<br>after 12 hours	**Escherichia coli- Milk:** MOI 100 at 4°C:<br>Reduction: 0.9 log10 CFU/mL<br>after 12 hours<br>MOI 1000 at 4°C:<br>Reduction: 1.7 log10 CFU/mL<br>after 12 hours<br>MOI 100 at 25°C:<br>Reduction: 4.2 log10 CFU/mL<br>after 12 hours<br>MOI 1000 at 25°C:<br>Reduction: 5.0 log10 CFU/mL<br>after 12 hours<br>**Salmonella- Milk:** MOI 100 at 4°C:<br>Reduction: 0.9 log10 CFU/cm²<br>after 12 hours<br>MOI 1000 at 4°C:<br>Reduction: 1.1 log10 CFU/cm²<br>after 12 hours<br>MOI 100 at 25°C:<br>Reduction: 1.4 log10 CFU/cm²<br>after 12 hours<br>MOI 1000 at 25°C:<br>Reduction: 1.5 log10 CFU/cm²<br>after 12 hours
**Combined effects of Salmonella phage cocktail and organic acid for controlling Salmonella Enteritidis in chicken meat**	Thailand	Salmonella spp.	10^5 CFU/mL	vB_SenM_P7 and vB_SenP_P32	MOI 100 - 10^7 PFU/mL	Effective against multiple Salmonella serovars	Phage survival >70% and >80% in propionic acid (0.25% v/v) at both temperatures; not observed in lactic acid at both temperatures after 24 hours	~ 70% adsorption within 5 minutes	0, 4, 8, 12, 24, and 48 hours	Poultry meat	Other	**At 4°C**:<br>**Phage Cocktail (MOI 100):** Reduction of 0.4 log CFU/g.<br>**Propionic Acid (0.25%):** Reduction of 1.2 log CFU/g.<br>**Combined Treatment:** Reduction of 1.4 log CFU/g.	
**Control of Escherichia coli O157:H7 using lytic bacteriophage and lactic acid on marinated and tenderized raw pork loins**	USA	Escherichia coli	10^6 CFU/mL	Lytic bacteriophage PhageGuard® S (Micreos Food Safety)	1 × 10^8 PFU/mL	Specific to E. coli O157	Stable at refrigeration temperatures	N/A	1 hour	Meat	Phage application & Phage application with sustances	**Phage application:** Pre-tenderized:<br>Phage 5%: Reduction of 1.90 logs<br>Post-tenderized:<br>Phage 5%: Reduction of > 2.50 logs<br>**Phage application with sustances:** Pre-tenderized:<br>LA 2.5% + Phage 5%: Reduction of 1.89 logs.<br>Post-tenderized:<br>LA 2.5% + Phage 5%: Reduction of > 1.75 logs.	

**Control of Listeria monocytogenes in milk by using phage cocktail**	Turkey	Listeria spp.	10^6 CFU/mL	Listex P100	10^8 PFU/mL	Effective against multiple Listeria monocytogenes strains	Effective at both 4°C and 30°C	N/A	1, 2, 3, 4, and 5 days	Milk	Phage cocktail application	**Milk:**<br>Reduction at 30°C after 4 days:<br>PL2: 1.9 log units<br>PL3: 2.5 log units<br>PL9: 2.9 log units<br>PL10: 2.7 log units<br>Reduction at 4°C after 4 days:<br>PL2: 1.0 log units<br>PL3: 1.6 log units<br>PL9: 1.0 log units<br>PL10: 1.4 log units
**"Control of Salmonella in Chicken Meat by a Phage Cocktail in Combination with Propionic Acid and Modified Atmosphere Packaging"**	Thailan d	Salmonella spp.	10^4 CFU/g	vB_SenM_P7 and vB_SenP_P32	10^8 PFU/mL	Effective against multiple serovars of Salmonella	Stable from 4°C to 70°C, pH 2 to 12	N/A	0, 1, 2, 3, 4, and 5 days	Poultry meat	Others	**Chicken Meat:**<br>Day 1 (all): 0.6 to 1.7 log<br>Day 2 (phage cocktail + PA (0.25% or 0.5%): 100% (4 log units)<br>Day 4 (PA-treated groups): complete reduction
**Development of a broad-spectrum Salmonella phage cocktail containing Viunalike and Jerseylike viruses isolated from Thailand**	Thailan d	Salmonella spp.	10^3 CFU/mL	ST-W77 and SE-W109	MOIs 10^3, 10^5, and 10^7	Broad host range against Salmonella strains	Stable at temperatures up to 40°C, retains infectivity at pH 4-11	N/A	Up to 7 days	Milk & Poultry meat	Phage cocktail application	**Milk:**<br>MOI 10^3: Slight decrease in bacterial growth after 24 hours, no difference compared to control after 48 hours<br>MOI 10^5: No viable Salmonella detected after 6 hours<br>MOI 10^7: No viable Salmonella detected after 6 hours (approximately 3 log reduction), sustained for 7 days<br>**Chicken Meat:** At 4°C:<br>MOI 10^8 PFU/cm^2: No viable Salmonella detected from 15 minutes up to 7 days
**Development of new strategy combining heat treatment and phage cocktail for post-contamination prevention**	Korea	Salmonella spp.	10^6 CFU/mL (Milk), 10^4 CFU/mL (Poultry meat)	TS3, TS6, TS13	10^7 PFU/mL (Milk), 10^8 PFU/mL (Poultry meat)	Effective against multiple Salmonella serovars	Stable at 70°C for 30 minutes	N/A	0, 6, 12, 24, and 48 hours	Milk & Poultry meat	Phage application with thermal processing	**Milk at 25°C:**<br>5.2–6.1 log CFU/mL reduction after 24 hours<br>Complete inhibition of bacterial growth up to 48 hours at refrigeration conditions<br>**Chicken Breast at 4°C:**<br>3.0–3.2 log CFU/cm^3 reduction after 6 hours<br>**at 25°C**:<br>4.2–4.5 log CFU/cm^3 reduction after 6 hours
**Effective control of Shigella contamination in different foods using a novel six-phage cocktail**	China	Other	10^8 CFU/mL	vB_SflS-ISF001, vB_SsoS-ISF002, vB_SdyS-ISF003, vB_SflM_004,	MOI 10^4 - 10^8 PFU/mL	Effective against multiple Shigella strains	Stable at 4°C and 25°C	N/A	0, 1, 2, 4, and 7 days	Poultry meat & Dairy products	Phage cocktail application	**Chicken Meat:**<br>Day 1: Reduction of 4.0 log CFU/g.<br>Day 2, 4, and 7: Reduction of 3.9 log CFU/g<br>**Yogurt:**<br>Day 1: Reduction of 3.4 log CFU/g.<br>Day 2: Reduction of 3.5 log CFU/g.<br>Day 4: Reduction of 3.6 log CFU/g.<br>Day 7: Reduction of 3.7 log CFU/g.

				vB_SdyM_006 , vB_SsoS_008										
**Efficacy of phage P100 on L. monocytogenes in refrigerated vacuum packaged cooked ham**	Belgium	Listeria spp.	~ 5 CFU/cm 2, corresponding to circa 15 CFU/g	Listex P100	1×10^7 and 5×10^7 PFU/cm²	Specific to Listeria monocytogenes	Effective at refrigeration temperatures	N/A	0, 14, 21, 28, 42, 59, 92, and 120 days	Meat	Phage application	**Cooked Ham: Sample 1**<br>**1x10^7 PFU/g**<br>Day 1: <1 CFU/g.<br>Day 14:1.0 x 10^0<br>Day 21: <1 CFU/g.<br>Day 28: <1 CFU/g.<br>Day 42: 2.4x10^1 CFU/g.<br>Day 59: 6.6x10^4 CFU/g.<br>Day 92: 1.3x10^6 CFU/g.<br>Day 120: 7.0x10^6 CFU/g.<br>**5x10^7 PFU/g:**<br>Day 1: <1 CFU/g.<br>Day 14: <1 CFU/g.<br>Day 21:<1 CFU/g.<br>Day 28:<1 CFU/g.<br>Day 42: <1 CFU/g.<br>Day 59: 2.1x10^4 CFU/g.<br>Day 92: 2.2x10^6 CFU/g.<br>Day 120: 5.2x10^6 CFU/g.	**Sample 2:**<br>1 x 10^7 PFU/g<br>Day 1: 1.0 x 10^0<br>Day 14: <1<br>Day 21: <1<br>Day 28: <1<br>Day 42: 1.0 x 10^1<br>Day 59: 3.4 x 10^5<br>Day 92: 7.0 x 10^2<br>Day 120: 6.6 x 10^7<br>**5 x 10^7 PFU/g**<br>Day 1:<1<br>Day 14:<1<br>Day 21:<1<br>Day 28:<1<br>Day 42:<1<br>Day 59: 6.8 x 10^1<br>Day 92 :8.1 x 10^4<br>Day 120: 1.8 x 10^8	**Sample 3:**<br>**1 x 10^7 PFU/g:**<br>Day 1: <1<br>Day 14:<1<br>Day 21:<1<br>Day 28:<1<br>Day 42: 7.2 x 10^1<br>Day 59: 5.0 x 10^1<br>Day 92: 1.2 x 10^7<br>Day 120: 7.8 x 10^3<br>5 x 10^7 PFU/g<br>Day 1: 1.0 x 10^0<br>Day 14: <1<br>Day 21: 1.0 x 10^0<br>Day 28: <1<br>Day 42: 1.0 x 10^0<br>Day 59: <1<br>Day 92: 7.4 x 10^6<br>Day 120: 4.3 x 10^3
**Enabling Cost-Effective Screening for Antimicrobials against Listeria monocytogenes in Ham**	USA	Listeria spp.	4.25 log CFU/g	Listex P100	2×10^7 PFU/g	Specific to Listeria monocytogenes	N/A	N/A	0 and 12 days	Meat	Phage application with substances	50 ppm of Ni, LM on sliced ham declined to 2.0 +- 0.20 log CFU/g, and LM on the MHM declined to 2.23 +- 0.40 log CFU/g		
**Engineered lytic phage of Bacillus cereus and its application in milk**	China	Bacillus spp.	10^5 CFU/mL	DK1 (Δgp36 DK1 after modification)	10^6 PFU/mL	Effective against Bacillus cereus strains 233-1 and 1582-3B	Stable at 4°C and 25°C for 7 days	Over 90% adsorption within 5 minutes	Evaluated over 12 hours	Milk	Others	In Milk:<br>After 3 hours: Reduction of 5.4 log10 CFU/mL for DK1 and 4.8 log10 CFU/mL for Δgp36 DK1<br>After 6 hours: Reduction maintained at around 5.2 log10 CFU/mL for DK1 and 5.5 log10 CFU/mL for Δgp36 DK1		

**Environmental conditions and serotype affect Listeria monocytogenes susceptibility to phage treatment in a laboratory cheese model**	USA	Listeria spp.	10^5 CFU/g	ListShield (Intralytix, Baltimore, MD) - a commercial phage cocktail	8×10^6 or 9×10^6 PFU/cm²	Effective against Listeria monocytogenes strains: 10403S (serotype 1/2a), FSL R9-5621, FSL R9-5623, FSL R9-5624, FSL R9-5625	Stable across various temperatures (6°C, 14°C, 22°C) and pH levels (5.5, 6.0, 6.5)	N/A	Evaluated over 1, 7, and 14 days	Dairy products	Phage application	Temperature Effects:<br>6°C: Average L. monocytogenes counts with phage: 6.33 log CFU/g;<br>14°C: Average L. monocytogenes counts with phage: 4.85 log CFU/g (day 1); 7.57 log CFU/g (day 14)<br>22°C: Significant reduction at higher temperatures<br>pH Effects:<br>pH 5.5: No significant growth of L. monocytogenes<br>pH 6.0: Phage-treated cheese had consistently lower L. monocytogenes counts compared to untreated cheese<br>pH 6.5: More effective phage treatment with significant reductions
**Escherichia coli phage phi2013: genomic analysis and receptor identification**	China	Escherichia coli	10^7 - 10^8 CFU/mL (depending on the specific experiment)	Phage phi2013	MOIs of 1 or 10	Effective against Escherichia coli laboratory strains (DH5α, M15, TG1, and S17-1λpir)	Temperature:<br>Survival rate of 67.0% at 50°C<br>Only 1.1% survival at 60°C<br>Trace amounts of infectivity at 70°C<br>pH:<br>Stable at pH 5.0 and pH 9.0<br>16.4% survival at pH 12.0<br>Trace levels of infectivity at pH 3.0	N/A	24 hours	Milk & Meat	Phage application	**Milk** at 25°C: No significant difference in killing efficiency between MOIs of 1 and 10<br>**Beef:** 4°C:<br>MOI of 1: Higher bacterial cell destruction compared to MOI of 10<br>Progeny virus produced more at MOI of 1<br>25°C:<br>Higher killing efficiency compared to 4°C
**Evaluation of an WPC edible film added with a cocktail of six lytic phages against foodborne pathogens such as enteropathogenic and Shigatoxigenic Escherichia coli**	Argentina	Escherichia coli	2-3 log CFU/mL	Cocktail of six phages: DT1 to DT6	5.2×10^7 PFU/disk	Effective against E. coli strains DH5α, EPEC, non-O157 STEC, and O157 STEC	Stable at 4°C and 24°C over 5 weeks	N/A	24 hours at 4°C, 24°C, and 37°C	Meat	Phage application with coating materials	Meat:<br>Phage-added WPC films:<br>Reduction at 25°C after 24 hours: E. coli cells reduced to non-detectable levels<br>Phage-added WPC films:<br>Reduction at 4°C after 24 hours: Complete inactivation achieved for DH5α and O157 STEC strains
**Examination of the Use of Bacteriophage as an Additive and Determining Its Best Application Method to Control Listeria monocytogenes in a Cooked-**	Canada	Listeria spp.	10^3-10^4 CFU/g	A511	~10^9 PFU/g	Effective against Listeria monocytogenes	Stable at 4°C over 28 days	N/A	Evaluated over 28 days	Meat	Phage application & Phage application with thermal processing	**Scenario 1**: **Both phage and bacteria inoculated in meat slurry before cooking**-Significant growth observed, indicating insufficient reduction by the phage treatment<br>**Scenario 2: Phage inoculated in meat slurry before cooking and then surface inoculated with bacteria**- Significant growth observed, indicating phage treatment was not effective when bacteria were surface inoculated<br>**Scenario 3: Bacteria inoculated in meat slurry before cooking and then surface inoculated with phage-** Initial 5-log reduction due to heat treatment; bacteria counts increased to >8-log CFU/g post heat treatment<br>**Scenario 4: Both phage and bacteria surface inoculated on cooked**

**Meat Model System**												**meat slurry**- Initial reduction by ~1.5 log CFU/g within 30 min, below detection limits within 24 hours, regrowth by day 7, significant increase by day 10
**"In-milk inactivation of Escherichia coli O157 by the environmental lytic bacteriophage ECPS-6"**	Poland, Germany	Escherichia coli	10^5 CFU/mL	ECPS-6	MOI 5 (filtered) and 50 (unfiltered milk)	Effective against Escherichia coli O157	Stable at 4°C, 20°C, and 37°C; reduced activity at 50°C and 60°C; highly reduced at 70°C	More than 99% of phage particles adsorbed within 8 minutes	10 days at 25°C and 4°C	Milk	Phage application	**Scenario 1:** E. coli O157<br>A-2 in Filtered Milk at MOI 5- 25°C<br>Reduction after 24 hours: 4.1 log CFU/ml<br>Reduction after 10 days: 0.7 log CFU/ml (compared to 7.3 log CFU/ml in control)<br>**Scenario 2:** E. coli O157<br>A-1 in Filtered Milk at MOI 5- 25°C<br>Reduction after 24 hours: 2.97 log CFU/ml<br>Regrowth after 10 days: 1.49 log CFU/ml (compared to 6.23 log CFU/ml in control)<br>**Scenario 3**: E. coli O157<br>A-2 in Filtered Milk at MOI 5- 4°C<br>Reduction after 10 days: 1.39 log CFU/ml<br>Reduction compared to control: 2.11 log CFU/ml<br>**Scenario 4**: E. coli O157<br>A-2 in Unfiltered Milk at MOI 50- 25°C<br>Reduction after 4 hours: 4.2 log CFU/ml<br>Reduction after 6 hours: Below detection limit<br>At 4°C<br>Reduction after 10 hours: 4.9 log CFU/ml compared to control
**Incorporation of A511 bacteriophage in a whey protein isolate-based edible coating for the control of Listeria monocytogenes in Cheese**	Mexico	Listeria spp.	7.0×10^7 CFU/g	A511	8.0×10 ^8 PFU/mL	Effective against Listeria monocytogenes	Stable at 4°C for 16 days	N/A	16 days	Dairy products	Phage application with coating materials & Others	**Phage-WPI Coating:**<br>Immediate reduction**:** 0.39 log CFU/g (5.6%) at zero-time<br>After 16 days at 4°C: 1.06 log CFU/g reduction compared to control<br>**Water Solution with Phages:**<br>Immediate reduction: 0.86 log CFU/g (12.4%) at zero-time<br>After 10 days: Reduction up to 0.16 log CFU/g compared to control
**Isolation and application of bacteriophages alone or in combination with nisin against planktonic and biofilm cells of Staphylococcus aureus**	Japan	Staphylococcus aureus	5 × 10^5 CFU/mL	SA46-CTH2	10^9 PFU/mL	Effective against Staphylococcus aureus	Stable up to 60°C, high stability from pH 5 to 10	Short latent period of 15 min, large burst size of 182 PFU/cell	24 hours	Milk	Phage application & Phage application with substances	**Phage application :** 2.2 log CFU/mL reduction after 24 hours (4°C)<br>**Phage application with substances:** 2.6 log CFU/mL at 24 h (4°C)

**Isolation and Characterization of a Novel Salmonella Phage vB_SalP_TR2**	China	Salmonella spp.	10^8 CFU/mL	vB_SalP_TR2	10^6 PFU/mL	High lytic potential against Salmonella serovars Albany, Corvallis, Newport, Kottbus, and Istanbul	Stable at 4-60°C, pH 4-11	N/A	0, 2, 4, 6, 8, 10, 12, and 24 hours	Milk & Poultry meat	Phage application	**Pasteurized milk**: 1.8 log at 6h-(37°C)<br>**Chicken meat:** 0.9 log at 6h - (37°C)
**Isolation and characterization of bacteriophages from soil against food spoilage and foodborne pathogenic bacteria**	Indonesia	Bacillus spp. & Escherichia coli	N/A	BC-S1 and BS-S2, Phage ETEC-S3, Phage EHEC-S4	Phages BC-S1 and BS-S2: miMOI of 10^-1 (pasteurized milk)<br>Phage ETEC-S3: miMOI of 10^-3 (chicken meat)<br>Phage EHEC-S4: miMOI of 1 (chicken meat)	Narrow host range	Stable at 4-60°C, pH 4-11	N/A	Overnight at 4°C and 28°C	Milk & Poultry meat	Phage application	**BC-S1**: pasteurised milk: 0.46 log (4 °C), 2.93 log (28 °C), BS-S2: pasteurised milk: 0.19 log (4°C), 1.07 log (28 °C)<br>**ETEC-S3:** chicken meat : 0.23 log (4 °C), 1.40 log (28 °C), **EHEC-S4:** chicken meat: 0.18 log (4 °C), 0.93 log (28 °C)
**Isolation and characterization of multidrug-resistant Salmonella-specific bacteriophages and their antibacterial efficiency in chicken breast**	South Korea, USA	Salmonella spp.	10^6 CFU/mL	STP1, STP2,STP3,STP4	MOIs 10, 10^2, and 10^3 - 10^7, 10^8, and 10^9 PFU/mL	High specificity towards Salmonella spp.	STP-1: Stable at 70°C and 75°C for 1 hour, pH 5 to 11<br>STP-2 and STP-3: Stable up to 80°C<br>STP-4: Stable for 1 hour at 60°C and 65°C, active for 30 minutes at 70°C, STP-2, STP-3, and STP-4: Active within the alkaline range up to pH 12	~80% adsorption in under 5 minutes	1, 3, 5, 7 days	Poultry meat	Phage cocktail application	Day 1:<br>MOI 1,000: 0.92 log reduction<br>Day 3:<br>MOI 10: 0.8 log reduction<br>MOI 100: 1.0 log reduction<br>MOI 1,000: 1.0 log reduction<br>Day 5:<br>MOI 10: 5.74 ± 0.24 CFU/g<br>MOI 100: 5.32 ± 0.09 CFU/g<br>MOI 1,000: 5.14 ± 0.16 CFU/g<br>Day 7:<br>MOI 10: 4.90 ± 0.25 CFU/g<br>MOI 100: 4.83 ± 0.31 CFU/g<br>MOI 1,000: 4.62 ± 0.21 CFU/g
**Isolation and characterization of phage ISTP3 for bio-control application against drug-resistant Salmonella**	China	**Drug-resistant** Salmonella spp.	10^4 CFU/sample	ISTP3	MOIs 10^2 and 10^3 - 10^6 and 10^7 PFU/ sample	Broad host range, effective against all tested Salmonella strains	Stable at 30-60°C, pH 4-12	52% after 5 min, 97% after 15 min	1, 3, 6, and 12 hours	Poultry meat	Phage application	**Chicken breast:**<br>4°C:<br>MOI 100: 1.6 log CFU/cm² reduction after 12 hours<br>MOI 1,000: 1.8 log CFU/cm² reduction after 12 hours<br>25°C:<br>MOI 100: 2.1 log CFU/cm² reduction after 12 hours<br>MOI 1,000: 2.4 log CFU/cm² reduction after 12 hours

**Isolation and Characterization of Two Lytic Bacteriophages Infecting a Multi-Drug-Resistant Salmonella Typhimurium and Their Efficacy to Combat Salmonellosis in Ready-to-Use Foods**	Egypt, Saudi Arabia	**Multi-Drug-Resistant** Salmonella spp.	10^4 CFU/mL	SPHG1 and SPHG3	MOIs 10^2 and 10^3 - 10^6 PFU/mL and 10^7 PFU/mL	Broad host range, lysis of 86% of tested Salmonella serovars	Stable at 30-70°C, pH 4-12	80% after 30 min at 80°C, no viable phages after 60 min at 80°C	48 hours	Milk & Poultry meat	Phage cocktail application	**Milk:**<br>25°C:<br>MOI 1,000: Below detection limit (<1 CFU/100 μL) after 2 hours<br>MOI 100: Below detection limit after 12 hours<br>4°C:<br>MOI 1,000: Below detection limit after 6 hours<br>MOI 100: Below detection limit after 16 hours<br>**Chicken breast:**<br>25°C:<br>MOI 1,000: Below detection limit after 2 hours<br>MOI 100: Below detection limit after 6 hours<br>4°C:<br>MOI 1,000: Below detection limit after 12 hours<br>MOI 100: Below detection limit after 16 hours
**Isolation and characterization of Bacillus cereus bacteriophage DZ1 and its application in foods**	China	Bacillus spp.	10^3 CFU/mL	DZ1	MOI 10^3	Lyses 41% of tested Bacillus cereus strains	Stable at 4-55°C, pH 5-9	N/A	6, 12, 24, 36, 48, 72 hours	Milk	Phage application	Milk:<br>4.21 log10 CFU/mL reduction after 6 hours at 25°C<br>No B. cereus detected in subsequent cultures after 72 hours
**Isolation, characterization and application of bacteriophage PSDA-2 against Salmonella Typhimurium in chilled mutton**	China	Salmonella spp.	10^4 CFU/mL	PSDA-2	MOIs 1, 10^2, and 10^4	Specific to Salmonella Typhimurium	Stable at 30-70°C, loses activity at 90°C; stable at pH 3-10, loses activity at pH 1 and 12	80% after 30 min at 80°C, no viable phages after 60 min at 80°C	Up to 144 hours	Meat	Phage application	Chilled mutton:<br>MOI 1: 0.4 log CFU/mL reduction after 144 hours<br>MOI 100: 0.7 log CFU/mL reduction after 12 hours<br>1.7 log CFU/mL reduction after 144 hours<br>MOI 10,000:1.2 log CFU/mL reduction after 12 hours<br>2.1 log CFU/mL reduction after 144 hours
**Isolation, characterization and comparison of lytic Epseptimavirus phages targeting Salmonella**	China	Salmonella spp.	10^6 CFU/mL	vB_SalS_1-23, vB_SalS_3-29, vB_SalS_1-19, vB_SalS_1-29, vB_SalS_2-3, vB_SalS_9-29	MOIs 1 and 10^2	Broad host range for most tested Salmonella strains	Stable at 30-60°C, loses activity at 70°C; stable at pH 4-11, loses activity at pH 1 and 12	N/A	Up to 7 hours	Poultry meat & Eggs	Phage cocktail application	**Chicken breast meat:**<br>MOI 1: 2–4.08 log10 CFU/mL reduction after 7 hours<br>MOI 100: 4.48–5.67 log10 CFU/mL reduction after 7 hours<br>Ground chicken meat:<br>MOI 100: 4.48–5.67 log10 CFU/mL reduction after 7 hours<br>Chicken skin:<br>MOI 1: Reduced to <2 log10 CFU/mL after 7 hours<br>MOI 100: Significant reduction (specific log reduction not provided)<br>**Eggshells:**<br>MOI 1: Reduced to 1 log10 CFU/mL after 7 hours<br>MOI 100: Below detection limit (specific log reduction not provided)

**Isolation, characterization, and application of lytic bacteriophages for controlling Enterobacter cloacae complex (ECC) in pasteurized milk and yogurt**	Egypt	Other	10^5 CFU/mL (Milk), 2 × 10^3 CFU/mL (Dairy products)	vB_EclM-EP1 and vB_EclM-EP2	10^7 PFU/mL	Effective against ECC isolates (6AS1, 3AS2, 8CS3), weak lysis for 2AS4; also effective against Aeromonas hydrophila and Klebsiella pneumoniae	vB_EclM-EP1: Stable at pH 3-9, inactivated at pH 11. Stable at 25°C and 37°C, reduced viability at 45°C and 55°C, completely inactivated at 70°C for 90 min or 80°C for 30 min vB_EclM-EP2: Stable at pH 5-11. Stable at 4-45°C, reduced viability at higher temperatures	N/A	Up to 6 days	Milk & Dairy products	Phage application	**Pasteurized Milk:**<br>At 4°C, 25°C, and 37°C:<br>vB_EclM-EP1: Complete inhibition after 4 days<br>vB_EclM-EP2: Complete inhibition after 3 days<br>Phage cocktail (vB_EclM-EP1 and vB_EclM-EP2): Complete inhibition after 3 days<br>**Yogurt:**<br>At 4°C:<br>vB_EclM-EP1: 2.5 log10 CFU/g reduction after 6 days<br>vB_EclM-EP2: 3 log10 CFU/g reduction after 6 days<br>Phage cocktail (vB_EclM-EP1 and vB_EclM-EP2): 3 log10 CFU/g reduction after 6 days<br>At 25°C:<br>vB_EclM-EP1: 1.3 log10 CFU/g reduction after 6 days<br>vB_EclM-EP2: 1.7 log10 CFU/g reduction after 6 days<br>Phage cocktail (vB_EclM-EP1 and vB_EclM-EP2): 2.3 log10 CFU/g reduction after 6 days<br>At 37°C:<br>vB_EclM-EP1: 1.5 log10 CFU/g reduction after 6 days<br>vB_EclM-EP2: 2 log10 CFU/g reduction after 6 days<br>Phage cocktail (vB_EclM-EP1 and vB_EclM-EP2): 2.5 log10 CFU/g reduction after 6 days
**Isolation, characterization, molecular analysis and application of bacteriophage DW-EC to control Enterotoxigenic Escherichia coli on various foods**	Indonesi a	Escherichia coli	10^6 CFU/mL	DW-EC	MOI 10^2	Effective against EHEC and EPEC with high EOP (>0.5)	Stable at 4°C for 21 weeks with 37.25% activity reduction	N/A	Up to 6 days	Poultry meat & Seafood	Phage application	**Chicken meat:**<br>80.93% reduction after 1 day<br>87.29% reduction after 6 days<br>**Fish meat:**<br>63.78% reduction after 1 day<br>87.89% reduction after 6 days
**Lytic bacteriophages UFJF_PfDIW6 and UFJF_PfSW6 prevent Pseudomonas fluorescens growth in vitro and the proteolytic-caused spoilage of raw milk during chilled storage**	Brazil	Other	10^3 CFU/mL	UFJF_PfDIW6 and UFJF_PfSW6	10^7 PFU/mL	Highly specific to the host bacterium	Stable at pH 5-11 and 63-72°C for 30 min; inactivated at pH 2 and by sodium hypochlorite	N/A	7 days	Milk	Phage application	Raw milk:<br>2.9 log CFU/mL reduction in total bacteria<br>2.5 log CFU/mL reduction in psychrotrophic bacteria<br>3.2 log CFU/mL reduction in Pseudomonas spp.

**Phage Biocontrol Improves Food Safety by Significantly Reducing the Level and Prevalence of Escherichia coli O157 in Various Foods**	USA	Escherichia coli	10^3 CFU/g	EcoShield PX (containing phages ECML-117, ECML-359, and ECML-363)	1×10^6, 5×10^6, 1×10^7 PFU/g	Effective against E. coli O157 , other big seven STEC serotypes	N/A	N/A	1, 24, 96, and 120 hours	Meat, Poultry meat, Seafood & Dairy products	Phage application	**Beef chuck roast:**<br>1×10^6 PFU/g: 0.2 log reduction<br>5×10^6 PFU/g: 0.4 log reduction<br>1×10^7 PFU/g: 0.5 log reduction<br>**Ground beef:**<br>5×10^6 PFU/g: 0.1 log reduction<br>1×10^7 PFU/g: 0.2 log reduction<br>**Raw chicken breast:**<br>5×10^6 PFU/g: 0.5 log reduction<br>1×10^7 PFU/g: 0.7 log reduction<br>**Cooked chicken:**<br>5×10^6 PFU/g: 0.4 log reduction<br>1×10^7 PFU/g: 0.7 log reduction<br>**Salmon:**<br>5×10^6 PFU/g: 0.4 log reduction<br>1×10^7 PFU/g: 0.7 log reduction<br>**Cheddar cheese:**<br>1×10^6 PFU/g: 0.4 log reduction<br>5×10^6 PFU/g: 1.3 log reduction<br>1×10^7 PFU/g: 1.5 log reduction
**Phage controlling method against novel freshwater-derived Vibrio parahaemolyticus in ready-to-eat crayfish (Procambarus clarkii)**	China	Other	10^5 CFU/mL	LPVP (containing phages LPVP16, LPVP19, and LPVP28)	MOIs 10^2 and 10^3	Effective against V. parahaemolyticus, no specific host range study details provided	N/A	N/A	Up to 12 hours	Seafood	Phage cocktail application	**Crayfish meat:**<br>At 4°C:<br>MOI 100:<br>Reduction after 4 hours: 0.95 log CFU/mL<br>Reduction after 6 hours: 0.99 log CFU/mL<br>MOI 1000:<br>Reduction after 4 hours: 0.95 log CFU/mL<br>Reduction after 6 hours: 2.22 log CFU/mL<br>At 25°C:<br>MOI 100:<br>Reduction after 4 hours: 1.01 log CFU/mL<br>Reduction after 6 hours: 1.39 log CFU/mL<br>MOI 1000:<br>Reduction after 2 hours: 0.41 log CFU/mL<br>Reduction after 4 hours: 1.16 log CFU/mL<br>Reduction after 6 hours: 2.36 log CFU/mL
**Reducing Salmonella enterica serovar Enteritidis contamination in food: lytic bacteriophages in a homemade mayonnaise-like matrix**	Chile, Spain	Salmonella spp.	10^3 CFU/mL	Cocktail of five phages	MOI 10^5	Effective against nine different SE strains	Stable at 4-18°C for 10 days	N/A	24 and 72 hours	Eggs	Phage cocktail application	**Homemade mayonnaise matrix**<br>24 hours:: 2.14 log reduction<br><br>72 hours: 3.75 log reduction

**Reduction of Nontyphoidal Salmonella enterica in Broth and on Raw Chicken Breast by a Broad-spectrum Bacteriophage Cocktail**	Canada	Salmonella spp.	4.0 × 10^4 CFU/g	SE4, SE13, and SE20	MOI 10^3 - 8.0 × 10^7 PFU/g	Broad-spectrum Effective against Enteritidis, Typhimurium, and Kentucky	N/A	N/A	Up to 5 days	Poultry meat	Phage cocktail application	**Raw chicken breast:**<br>10°C:<br>1.65 log reduction after 0 days<br>3.32 log reduction after 1 day<br>3.21 log reduction after 5 days<br>22°C:<br>1.02 log reduction after 0 hours<br>1.09 log reduction after 4 hours<br>1.20 log reduction after 8 hours<br>1.71 log reduction after 16 hours
**Reduction of Salmonella contamination on the surface of chicken skin using bacteriophage**	UK	Salmonella spp.	10^8 CFU/mL	Eϕ151, Tϕ10, Tϕ11	10^9 PFU/ml	N/A	N/A	N/A	Up to 24 hours	Poultry meat	Phage cocktail application	Chicken skin:<br>S. Enteritidis: 1.38 log10 MPN reduction<br>Reduction Range: 0.95 to ≥3.04 log MPN per skin section<br>S. Typhimurium: 1.83 log10 MPN reduction<br>Reduction Range: 1.46 to ≥3.04 log MPN per skin section
**Sterilizing effect of phage cocktail against Shiga toxin-producing Escherichia coli O157 in foods**	China	Escherichia coli	10^3 CFU/mL (Milk), 10^3 CFU/cm ^3 (Meat)	PNJ1902, PSD2001, PSD2002	10^6 PFU/mL or 10^6 PFU/cm^2	Broad host range, effective against 20 out of 30 E. coli O157 strains and 20 non-O157 E. coli strains	N/A	N/A	Up to 12 hours	Milk & Meat	Phage cocktail application	**Milk:**<br>7 log reduction at 24°C and 37°C for 12 hours<br>**Raw beef:**<br>4 log reduction at 24°C for 12 hours<br>5 log reduction at 37°C for 12 hours
**Tackling Vibrio parahaemolyticus in ready-to-eat raw fish flesh slices using lytic phage VPT02 isolated from market oyster**	Korea	Other	3 × 10^4 CFU/g	VPT02	MOI 10	Broad host range, effective against V. parahaemolyticus strains	Stable from 4°C to 50°C for 12 hours; stable at pH 3 to 11 for 12 hours	Quick adsorption within 15 minutes	Up to 6 hours	Seafood	Phage application	At 25°C:<br>1.5 log reduction in 6 hours at MOI of 10<br>2.0 log reduction in 6 hours at MOI of 10<br>3.9 log reduction overall
**Temperate phage influence virulence and biofilm-forming of Salmonella Typhimurium and enhance the ability to contaminate food product**	China	Salmonella spp.	10^4 CFU/mL	vB_Sal_PHB48	10^6 PFU/mL	Effective against multiple strains of Salmonella Typhimurium	Stable from 4°C to 50°C for 12 hours; stable at pH 3 to 11 for 12 hours	N/A	Up to 9 hours	Milk & Poultry meat	Phage application	**Milk:**<br>Sal013+: 8.66 × 10^9 CFU/mL after 9 hours<br>Sal013: 4.77 × 10^9 CFU/mL after 9 hours<br>**Chicken:**<br>Sal013+: 1.60 × 10^5 CFU/sample after 9 hours<br>Sal013: 1.22 × 10^5 CFU/sample after 9 hours
**The application of adaptively evolved thermostable bacteriophage**	Korea	Salmonella spp.	10^5 CFU/cm²	ΦYMFM0293	10^8 PFU/mL	Effective against Salmonella Typhimurium, Salmonella	Stable at 66°C for chicken skin and 68°C for duck skin for 4 hours;	Quick adsorption within 3 minutes	Up to 24 hours	Poultry meat	Phage application with	**Chicken skin:**<br>(1-log and 1.6-log CFU/cm2 after 1 and 3 h of storage, 2.0-log reduction n up to 24 h), more than 5-log

**ΦYMFM0293 to control Salmonella spp. in poultry skin**						Enteritidis, and other Salmonella spp.	stable at pH 3 to 11				thermal processing	**Duck skin:**<br>1.4-log CFU/cm2 at 24 h
**The application of the lytic domain of endolysin from Staphylococcus aureus bacteriophage in milk**	China	Staphylococcus aureus (including methicillin-resistant-MRSA)	10^8 CFU/mL	LysGH15 and CHAPLysGH15	25 nM of LysGH15 and CHAPLysGH15	Effective against Staphylococcus aureus and MRSA	LysGH15: Optimal at 35°C and pH 6.0; stable from pH 6.0 to 10.0 CHAPLysGH15: Optimal at 40°C and pH 9.0; stable from pH 6.0 to 10.0	N/A	8 hours	Milk	Phage application	The CHAPLysGH15 -MRSA 2701: ~ 2.5-log reduction in whole and skim milk after 8 hours at 4°C
**The Lytic Siphophage vB_StyS-LmqsSP1 Reduces the Number of Salmonella enterica Serovar Typhimurium Isolates on Chicken Skin**	Germany	Salmonella spp.	2 × 10^3 CFU/cm 2	vB_StyS-LmqsSP1	2.5 × 10^8 PFU/cm2	Effective against Salmonella enterica serovar Typhimurium	Stable at 4°C, reduced efficacy at 37°C,	N/A	7 days	Poultry meat	Others	Chicken skin:<br>(S1-S2-LT2) Approximately 1.8-log reduction after 3 hours<br>(S1 or S2) Approximately 2.2-log reduction after 24 hours,<br>(LT2) 1.9-log reduction
**The strategy of biopreservation of meat product against MRSA using lytic domain of lysin from Staphylococcus aureus bacteriophage**	China	Staphylococcus aureus (MRSA)	10^4 CFU/cm^2	LysGH15 and CHAPLysGH15	0.4 nmol/cm^2 of LysGH15 and CHAPLysGH15	Effective against multiple strains of S. aureus including MRSA	LysGH15: Optimal at 35°C and pH 6.0; stable from pH 6.0 to 10.0 CHAPLysGH15: Optimal at 40°C and pH 9.0; stable from pH 7.0 to 10.0	N/A	2 hours	Meat	Phage application	**Pork:**<br>0.4 nmol/cm^2 of CHAPLysGH15:<br>Reduction: Complete elimination of MRSA<br>0.4 nmol/cm^2 of LysGH15:<br>Reduction: 1.0 log CFU/cm^2<br>**Chinese bacon:**<br>0.4 nmol/cm^2 of LysGH15:<br>Reduction: Complete elimination of MRSA<br>0.2 nmol/cm^2 of LysGH15:<br>Reduction: 1.93 ± 0.24 log CFU/cm^2<br>0.1 nmol/cm^2 of LysGH15:<br>Reduction: 0.47 ± 0.12 log CFU/cm^2<br>0.4 nmol/cm^2 of CHAPLysGH15:<br>Reduction: 0.7 log CFU/cm^2
**Use of Cocktail of Bacteriophage for Salmonella Typhimurium Control in Chicken Meat**	Chile	Salmonella spp.	10^4 CFU/g	Phage cocktail (Siphoviridae family: A7, A8, B3; Microviridae family: A4, A5)	10^9 PFU/mL	Effective against Salmonella Typhimurium	N/A	N/A	24, 48, and 72 hours	Poultry meat	Phage cocktail application	At 22°C:<br>24 hours: 1 log10 CFU/g<br>Reduction after 48 hours: 1.7 log10 CFU/g<br>Reduction after 72 hours: 1.7 log10 CFU/g<br>At 30°C:<br>24 hours: 1.0 log10 CFU/g reduction<br>At 10°C:

												48 hours: 1.5 log10 CFU/g reduction<br>72 hours: 1.2 log10 CFU/g reduction
**Vacuum-Packed Steak Tartare: Prevalence of Listeria monocytogenes and Evaluation of Efficacy of ListexTM P100**	Czech Republi c	Listeria spp.	10^3 CFU/g	ListexTM P100	10^8 and 10^9 PFU/g	Effective against Listeria monocytogenes	N/A	N/A	1, 5, 24, 48, and 72 hours	Meat	Phage application	**Steak tartare:**<br>Sensitive Isolate LV1242 (10^9 PFU/g):<br>After 1 hour: Slight reduction observed<br>After 72 hours: No significant reduction compared to control<br>Resistant Isolate LV830 (10^9 PFU/g):<br>No significant reduction observed at any time point
**Virulent phage vB_CpeP_HN02 inhibits Clostridium perfringens on the surface of the chicken meat**	China	Other	10^4 – 5 × 10^4 CFU/g	vB_CpeP_HN 02	1 × 10^6 - 1 × 10^10 PFU/g	Effective against Clostridium perfringens	Stable from pH 5 to 11 and up to 70°C	45% adsorption within 30 minutes	48-72 hours	Poultry meat	Phage application	**Chicken meat:**<br>At 1 × 10^6 PFU/g:<br>Reduction after 72 hours: 2.63 log10 CFU/g<br>At 1 × 10^9 PFU/g:<br>Reduction after 72 hours: 3.61 log10 CFU/g<br>At 1 × 10^10 PFU/g:<br>Reduction after 48 hours: Complete lysis of C. perfringens
